# Supplementary material for: Bayesian estimation of predator diet composition from fatty acids and stable isotopes
Source: PeerJ. 2015 Apr 23;3:e920. doi: 10.7717/peerj.920 (PMC4411522; doi:10.7717/peerj.920)
Supplement: Supplemental Information 1 [file peerj-03-920-s001.pdf]

Simulated example: Estimating diet proportions form fatty acids and stable isotopes

Philipp Neubauer

Dragonfly Science,
PO Box 27535, Wellington 6141, New Zealand

June 11, 2014

1 Preamble

DISCLAIMER: This is an evolving package, please read instructions here for installation and to see what dependencies are required. If you find a bug or want so suggest improvements, please submit an issue, collaborations and contributions are very welcome!

DISCLAIMER 2: This tutorial is by no means a real analysis, which should proceed more carefully, and with longer MCMC runs. The example is designed to run through in a few minutes, and is therefore only an illustration of how an analysis of real data could proceed.

```
## Loading required package:  methods
```

```
library(fastinR, warn.conflicts = F)

## Loading required package:  rjags
## Loading required package:  coda
## Loading required package:  lattice
## Linked to JAGS 3.4.0
## Loaded modules:  basemod,bugs
```

2 Introduction

We start with a very simple simulated example. It is possible to simulate relevant data using the built in simulation GUI, which is called from the command line once fastinR has been loaded. Please type ?simulation to obtain help on simulating your own 'dataset'. Note, however, that the recommended way to

interact with the package is the command line - the GUI is somewhat unstable and has a mind of its own (see warnings in GUI help files).

3 Loading data from files

Raw data should be stored in .csv files in a prescribed format, see *add_FA*, *add_SI* and *add_Covs* for details on formatting. You can also inspect saved output from *simulation()* to get a better idea of the correct file format.

Files are imported with the help of three constructor functions: *add_FA*to import fatty acid data, *add_SI* to import stable isotope data and *add_Covs* to import data on covariates or groupings that may be influencing predator diets.

The package comes with a simulated dataset which can be called with , which loads an object called *datas* into the current environment.

The package also comes with the same simulated data in .csv files to illustrate the file format and load procedure. These .csv files can be found using the *system.file* function which finds package internal files. The *add_SI* constructor takes separate files for predator and prey stable isotope data, as well as files for additive fractionation coefficient means and variances - these are optional and can be specified manually in the function call, see the function help for details.

```
# these commands return paths to data supplied with
# the package. For a real analysis these would be
# substituted with paths to data files, or with R
# data objects.
SI.predators <- system.file("extdata", "Simdata_SI_preds.csv",
    package = "fastinR")
SI.preys <- system.file("extdata", "Simdata_SI_preys.csv",
    package = "fastinR")
Frac.Coeffs.mean <- system.file("extdata", "Simdata_SI_fc_means.csv",
    package = "fastinR")
Frac.Coeffs.var <- system.file("extdata", "Simdata_SI_fc_var.csv",
    package = "fastinR")

dats <- add_SI(SI.predators = SI.predators, SI.preys = SI.preys,
    Frac.Coeffs.mean = Frac.Coeffs.mean, Frac.Coeffs.var = Frac.Coeffs.var)
```

We can now visualize the data on a plot like so:

```
dataplot(dats)
```

The dats object now has a set of data in the right format for further analysis. We'll add fatty acids before we proceed. As with *add_SI*, the *add_FA* constructor takes separate files for predator and prey fatty acid data, conversion coefficient means and variances as well as fat content - these are again optional and can be specified manually in the function call, see the function help for details.

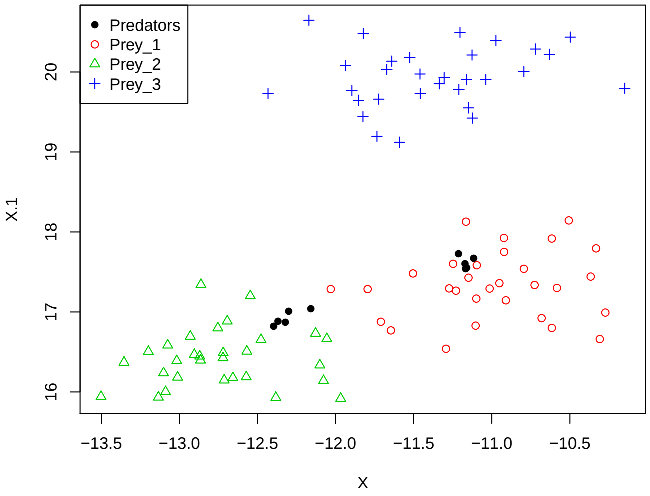

Figure 1: SI for simualted data

```
FAP.predators <- system.file("extdata", "Simdata_FA_preds.csv",
    package = "fastinR")
FAP.preys <- system.file("extdata", "Simdata_FA_preys.csv",
    package = "fastinR")
Conv.Coeffs.mean <- system.file("extdata", "Simdata_FA_cc_means.csv",
    package = "fastinR")
Conv.Coeffs.var <- system.file("extdata", "Simdata_FA_cc_var.csv",
    package = "fastinR")
fat.conts <- system.file("extdata", "Simdata_fat_cont.csv",
    package = "fastinR")

dats <- add_FA(FA.predators = FAP.predators, FA.preys = FAP.preys,
    fat.conts = fat.conts, Conv.Coeffs.mean = Conv.Coeffs.mean,
    Conv.Coeffs.var = Conv.Coeffs.var, datas = dats)
```

Note the last argument in *add_FA* now contains a reference to the data object that was constructed from the Stable Isotopes beforehand. *add_FA* just adds fatty acid data to the mix - the same would apply if data were added the other way around (only *add_Covs* works separately, it needs it's own object).

Plotting the joint dataset:

```
dataplot(dats)
```

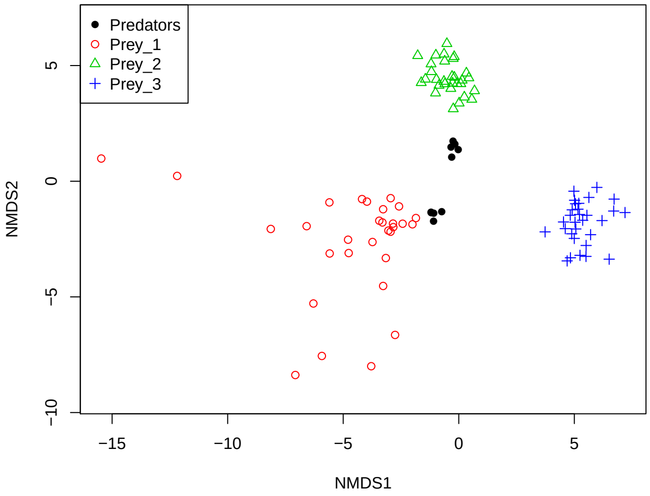

Figure 2: Combined dataset projected using NMDS

4 Data Grooming

The grooming step in this case corresponds to selecting relevant variables for the analysis. In practice, one could measure lots of Fatty Acids, and researchers often choose arbitrary cutoff points in proportions to reduce the dataset. However, even Fatty Acids occurring in low proportions may be useful for source discrimination and diet estimation if they introduce systematic differences between sources (prey items). The *select_vars* function provides a graphical way to choose a subset of Fatty Acids according to their contribution to prey separation and reduction in co-linearity in the prey matrix. Using *select_vars* in the simulated example shows that only really 2 Fatty Acids contribute to source separation - the cumulative separation displayed in the console converges to one after adding the two most important fatty acid proportions: all remaining FAs only add to co-linearity in the source matrix. We choose Fatty Acid no 3,2 and 6 for analysis (the second function call does this directly without prompting the user, by giving the index of the fatty acids to choose).

```
dats.subset <- select_vars(dats)
```

or

```
dats.subset <- select_vars(dats, c(3, 2, 6))

# inspecting
```

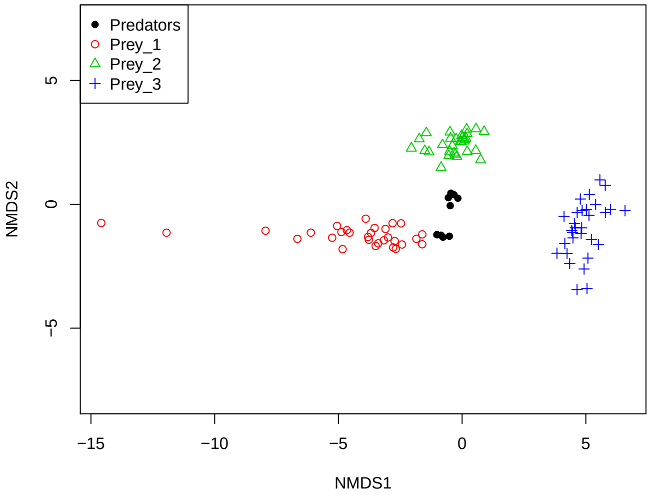

Figure 3: FA subset projected by NMDS

```
dats.subset$datas.FA$n.fats
```

The data object *dats* is now ready for analysis, we can plot the dataset with it's new subset of fatty acids as before:

```
dataplot(dats.subset)
```

Apart from a rotation, the overall configuration should be similar.

5 Bayesian Analysis

The actual analysis uses Markov Chain Monte Carlo to estimate posterior distributions for diet parameters. Estimation can be performed locally (in the active R session) or in a distributed way, using as many R sessions as Markov Chains. A good strategy is usually to run a few chains locally for short runs, and then run 3 or more chains in parallel in a distributed way once a satisfactory set of priors, thinning interval etc has been found (setting the number of chains to a maximum of n-1 cores on your CPU is a good idea to not hog ALL resources). The *run_MCMC* functions sets up the MCMC runs and takes *spawn*

= T or *spawn* = F as parameter to run chains in R slave processes or locally, respectively.

The Rnot parameter sets the prior scale for the predator logistic normal covariance matrix, and will often determine how well the chain mixes, that is, how well it explores the parameter space, or if it gets stuck in local modes (that can be of low probability). In the latter case, one would see strong auto-correlation in the Markov Chains for individual parameters. Increasing *Rnot* and/or *Rnot_SI* can help, but will make it harder to get precise estimates. There is thus a trade-off between accuracy and mixing here, and often one will just have to run an analysis for many iterations and with a large thinning interval (e.g., ¿100k iterations with a thinning interval of ¿100). This should be done after finding reasonable parameters for Rnot on a shorter run, and then letting the final analysis run with long chains.

For a combined analysis of stable isotopes and fatty acids, it is often useful to run the two data types separately to assure that good priors can be found for both datasets independently, and then combining them for a final analysis. The analysis type is chosen in the appropriate option in the function call.

5.1 Estimating population proportions

5.1.1 Stable Isotopes alone

Lets start with an analysis of the stable isotopes, estimating only global (population) level diets. We will use the default prior on the predator covariance matrix, and will adjust this prior subsequently. *WARNING* This might take a while depending on your resources, the size of the dataset and the parameters used for the MCMC.

```
Pop.SI <- run_MCMC(datas = dats.subset, nIter = 20000,
    nBurnin = 10000, nChains = 3, nThin = 20, Data.Type = "Stable.Isotopes",
    Analysis.Type = "Population.proportions", Rnot_SI = 0.1,
    plott = F, spawn = F)
```

Plotting the MCMC is the easiest way to ensure that the sampler is mixing - meaning that the chain explores the posterior distribution of each parameter efficiently.

```
MCMCplot(Pop.SI)
```

The mixing isn't great, meaning that some auto-correlation is evident in the Markov Chain time series in left-hand plots. The *diags* function gives more information, displaying two types of diagnostics in the console.:

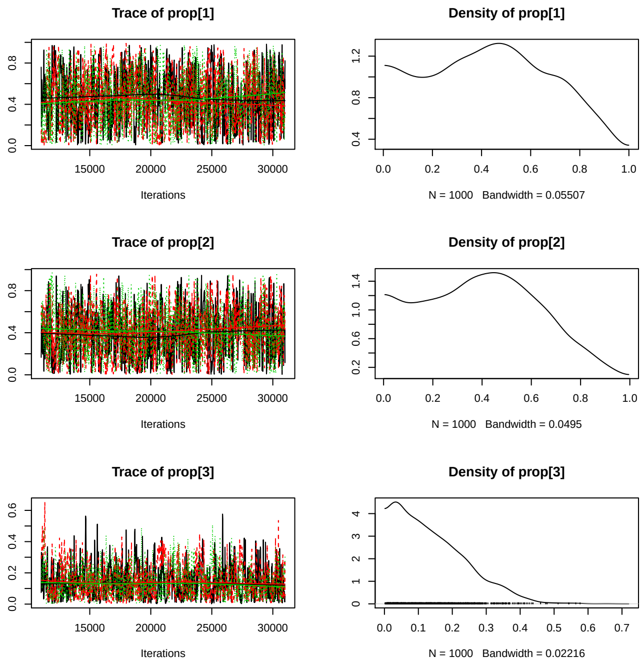

Figure 4: MCMC traces showing 3 individual Markov Chains (colours)

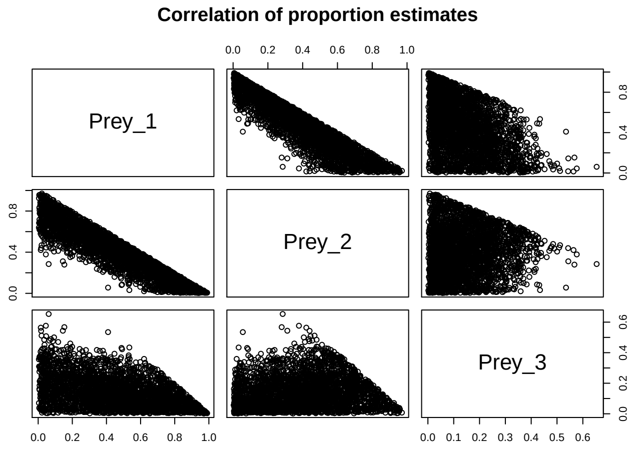

Figure 5: Posterior distributions for diet proportions from SI

```
diags(Pop.SI)
```

Based on the diagnostics, it seems that we're doing OK, but that the Stable Isotopes don't give much certainty about diet proportions:

```
summary(Pop.SI)
```

```
plot(Pop.SI, save = F)

## Loading required package:  grid
## Using  as id variables
```

The credible intervals are very large for Prey items 1 and 2, and the correlation plot suggests that there is a reasonably strong posterior correlation between estimates of prey items 1 & 2.

Increasing the default prior Rnot_SI on the predator covariance matrix sometimes leads to better mixing. We'll also run more iterations and set $spawn = T$ for this.

```
Pop.SI2 <- run_MCMC(datas = dats.subset, nIter = 1e+05,
    nBurnin = 10000, nChains = 3, nThin = 100, Data.Type = "Stable.Isotopes",
    Analysis.Type = "Population.proportions", Rnot_SI = 1,
    plott = F, spawn = T)
```

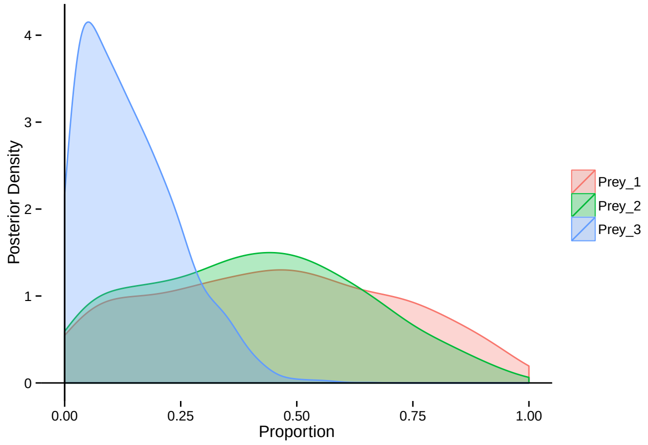

Figure 6: Posterior distributions for diet proportions from SI

```
MCMCplot(Pop.SI2)
```

```
diags(Pop.SI2)
```

Mixing only improves marginally, indicating that this may be good as it gets with stable isotopes alone.

5.1.2 Fatty Acids alone

We repeat this analysis with Fatty Acids, again starting with the default prior. Note that $spawn = T$ this time to save some time by calling 3 R processes at once - a recommendation is to use the number of cores of the computer -1. (to know how many cores you have, get the multicore package and use multicore:::detectCores(), or look at the number of CPUs displayed in your system monitor (resmon on windows))

```
Pop.FA <- run_MCMC(datas = dats.subset, nIter = 10000,
    nBurnin = 1000, nChains = 3, nThin = 10, Data.Type = "Fatty.Acid.Profiles",
    Analysis.Type = "Population.proportions", Rnot = 0.2,
    plott = F, spawn = T)
```

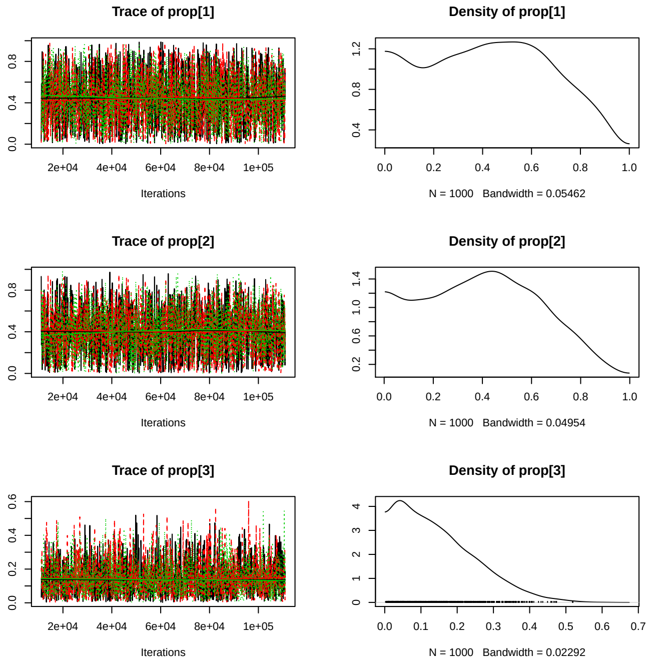

Figure 7: MCMC traces for second run with SI showing 3 individual Markov Chains (colours)

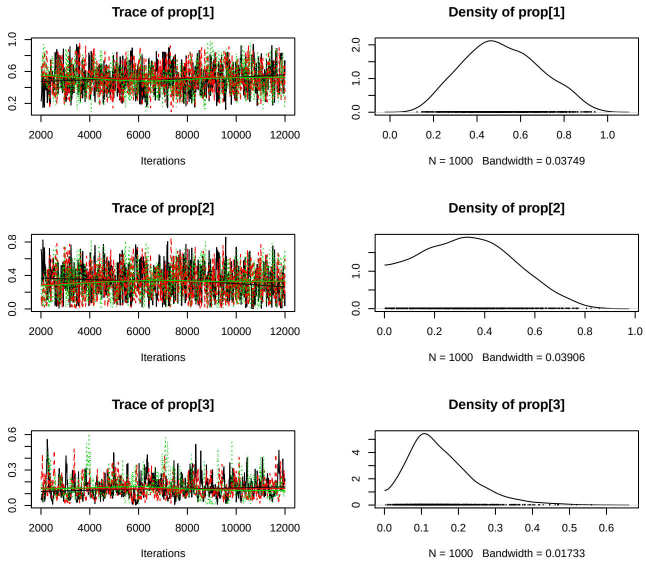

Figure 8: MCMC traces from FAP analysis showing 3 individual Markov Chains (colours)

```
MCMCplot(Pop.FA)
```

Once again the mixing isn't great:

```
diags(Pop.FA)
```

Again not too bad according to the diagnostics, although the chains should be run for longer (see Raftery-Lewis diagnostics) - but lets try again with *Rnot = 1*

```
Pop.FA2 <- run_MCMC(datas = dats.subset, nIter = 10000,
    nBurnin = 1000, nChains = 3, nThin = 10, Data.Type = "Fatty.Acid.Profiles",
    Analysis.Type = "Population.proportions", Rnot = 1,
    plott = F, spawn = T)
```

```
MCMCplot(Pop.FA2)
```

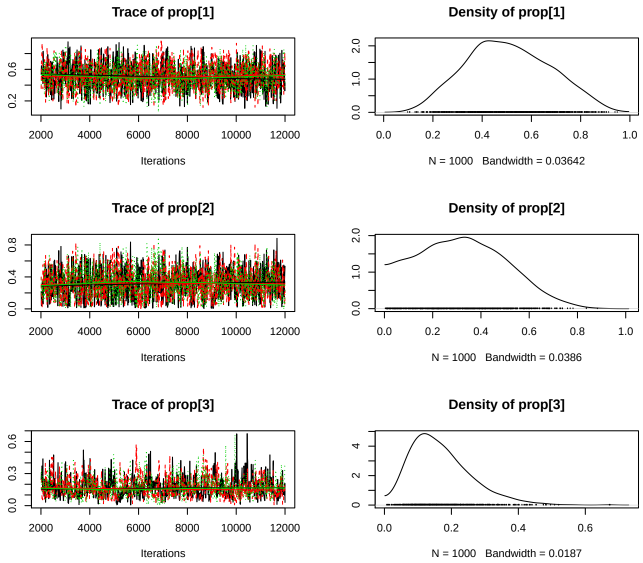

Figure 9: MCMC traces from the second run of the FAP analysis showing 3 individual Markov Chains (colours)

```
diags(Pop.FA2)
```

Looks very similar, so we do a final run with 30k iterations as suggested by the *diags* output and a thinning interval of 30 should be better. (Note that values suggested by the diagnostics may vary from one run to the next. Also note that we already ran 30k as we ran 3 parallel chains here...).

```
Pop.FA3 <- run_MCMC(datas = dats.subset, nIter = 30000,
    nBurnin = 10000, nChains = 3, nThin = 30, Data.Type = "Fatty.Acid.Profiles",
    Analysis.Type = "Population.proportions", Rnot = 1,
    plott = F, spawn = T)
```

```
MCMCplot(Pop.FA3)
```

```
diags(Pop.FA3)
summary(Pop.FA3)
```

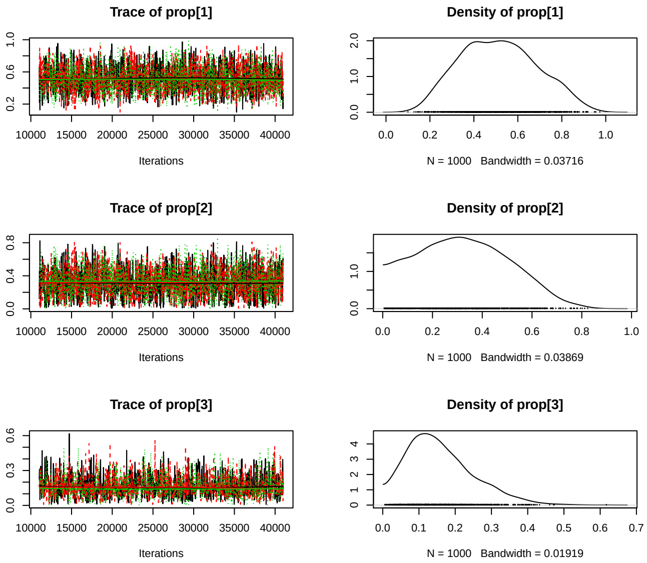

Figure 10: MCMC traces from the third run of the FAP analysis showing 3 individual Markov Chains (colours)

```
plot(Pop.FA3, save = F)

## Using  as id variables
```

There is still substantial uncertainty about the diet proportions: it looks as though Prey 1 is a dominant source, but the credible intervals are large. Combining stable isotopes and fatty acids to see if there is a gain in information:

5.1.3 Combining fatty acids and stable isotopes

```
Pop.Combined <- run_MCMC(datas = dats.subset, nIter = 30000,
    nBurnin = 10000, nChains = 3, nThin = 30, Data.Type = "Combined.Analysis",
    Analysis.Type = "Population.proportions", Rnot = 1,
    Rnot_SI = 1, plott = F, spawn = T)
```

```
MCMCplot(Pop.Combined)
```

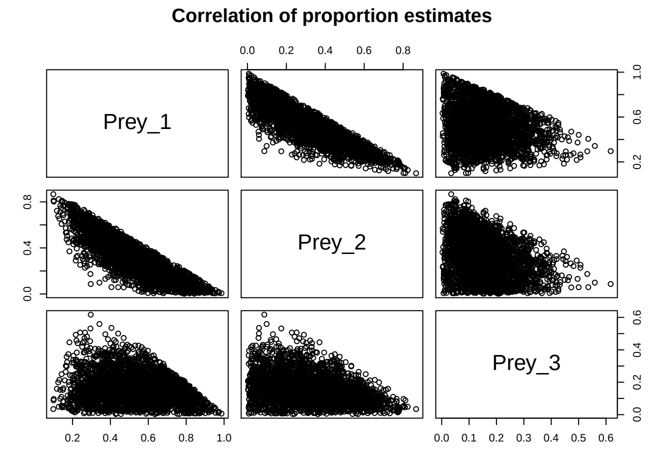

Figure 11: Posterior distributions for diet proportions from the FAP analysis

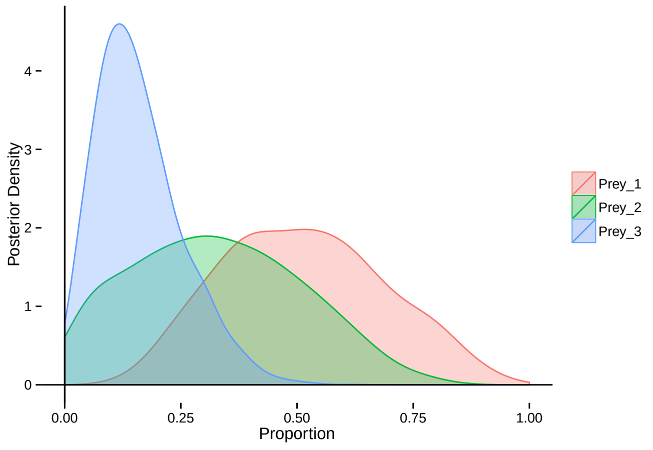

Figure 12: Posterior distributions for diet proportions from the FAP analysis

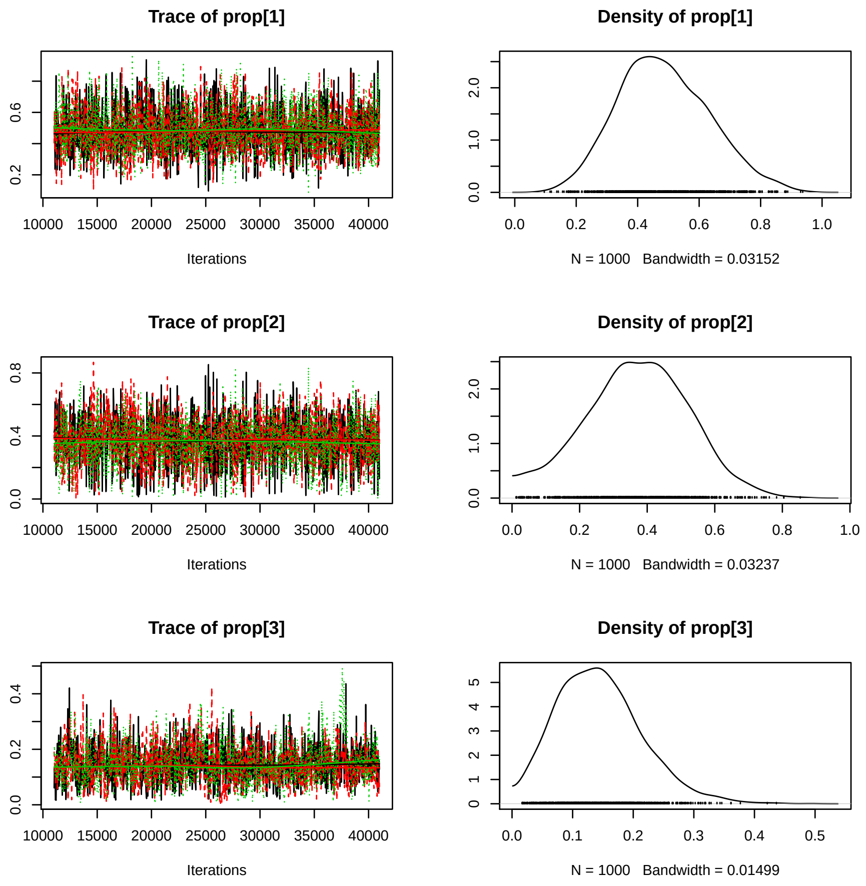


```
diags(Pop.Combined)
summary(Pop.Combined)
```

```
plot(Pop.Combined, save = F, types = "post")

## Using  as id variables
```

The combined analysis reduces uncertainty slightly, especially for source 3, but dietary sources remain uncertain with somewhat large credible intervals.

To compare the three approaches explicitly, we can use *multiplot*. For *multiplot* the three results need to be combined in a list:

```
Pop.list <- list(Stable.Isotopes = Pop.SI2, Fatty.Acids = Pop.FA3,
    Combined = Pop.Combined)
```

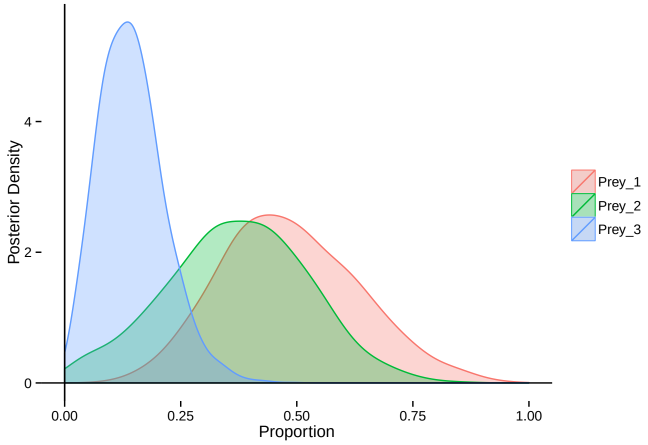

Figure 13: Posterior distributions for diet proportions from the combined analysis

```
multiplot(Pop.list, save = F, types = "violin")

## Using V1 as id variables
```

The original data was simulated to include groups of predators with rather different proportions (visible in the dataplots above). Taking a look at the proportions that were used to simulate the dataset:

```
proportions.path <- system.file("extdata", "Simdata_props.csv",
    package = "fastinR")
proportions <- read.csv(proportions.path, header = F,
    row.names = 1)

colnames(proportions) <- unique(dats$prey.ix)

# show simualted proportions
proportions



# overall mean proportions
colMeans(proportions)
```

Looking at the proportions, it is clear that there are two groups of predators (e.g., juveniles and adults), one group preys preferentially on Prey 1 while the

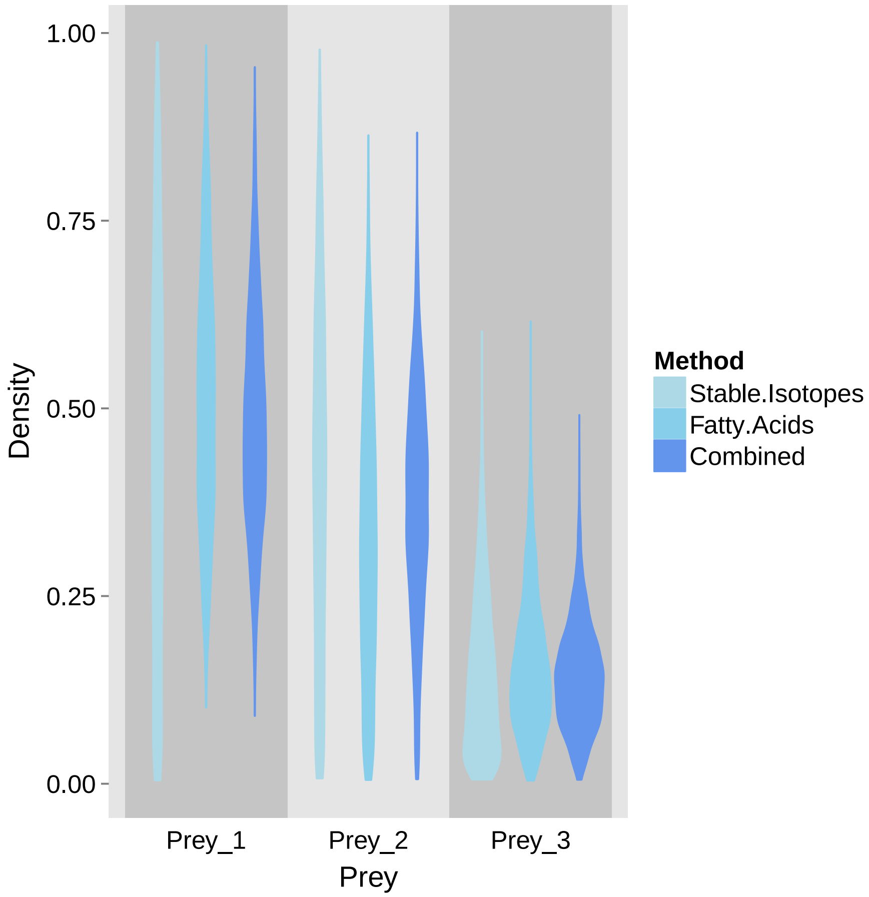

Figure 14: Comparing posterior distributions for diet proportions

other preys mostly on Prey 2. Let's see if we can pick this up by estimating individual predator proportions:

5.2 Estimating individual proportions

Lets try this with fatty acids first - given that there was limited information about diets in the stable isotopes alone, they can't be expected SI alone to provide the extra information we're after.

We now need to deal with one extra prior to be set: the prior for the variance of diet proportions. After some exploratory runs, *even=2* seems like a reasonable compromise between our ability to detect differences (facilitated for smaller values of *even*) and the ability of the Markov Chains to mix properly (easier for larger *even*- see the documentation of *run_MCMC*).

5.2.1 From Fatty Acids

```
Ind.FA <- run_MCMC(datas = dats.subset, nIter = 1e+05,
    nBurnin = 10000, nChains = 3, nThin = 10, Data.Type = "Fatty.Acid.Profiles",
    Analysis.Type = "Individual.proportions", Rnot = 1,
    even = 1, plott = F, spawn = T)
```

We won't show the output of the next commands anymore since it is far too long. The commands are the same as for a population proportion analysis:

```
MCMCplot(Ind.FA)

diags(Ind.FA)

summary(Ind.FA)
```

```
plot(Ind.FA, save = F, type = "post")

## Using  as id variables
## Using rep(i, nrow(outs)) as id variables
```

Despite this MCMC run being an order of magnitude too short, we can see that the grouped pattern emerges quite clearly in the last plot. Nevertheless, posterior correlations remain for prey items 1&2 remain strong (we'll soon see why!). First, let's see if the grouping becomes more pronounced with the inclusion of stable isotopes:

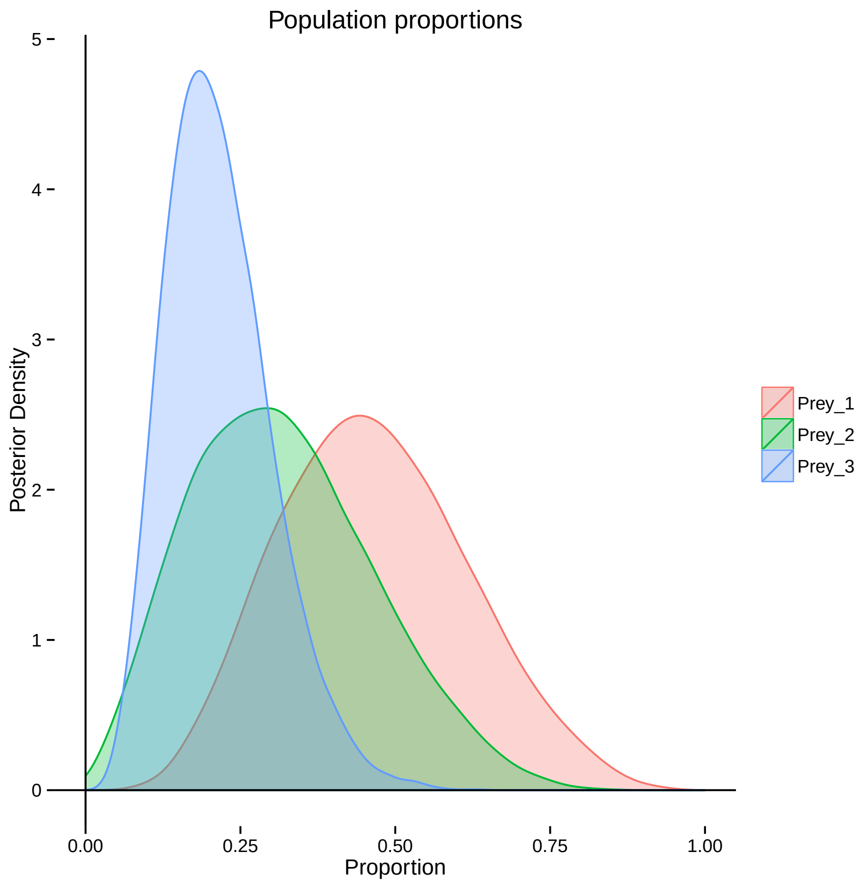

Figure 15: Posterior distributions for individual diet proportions from the FAP analysis

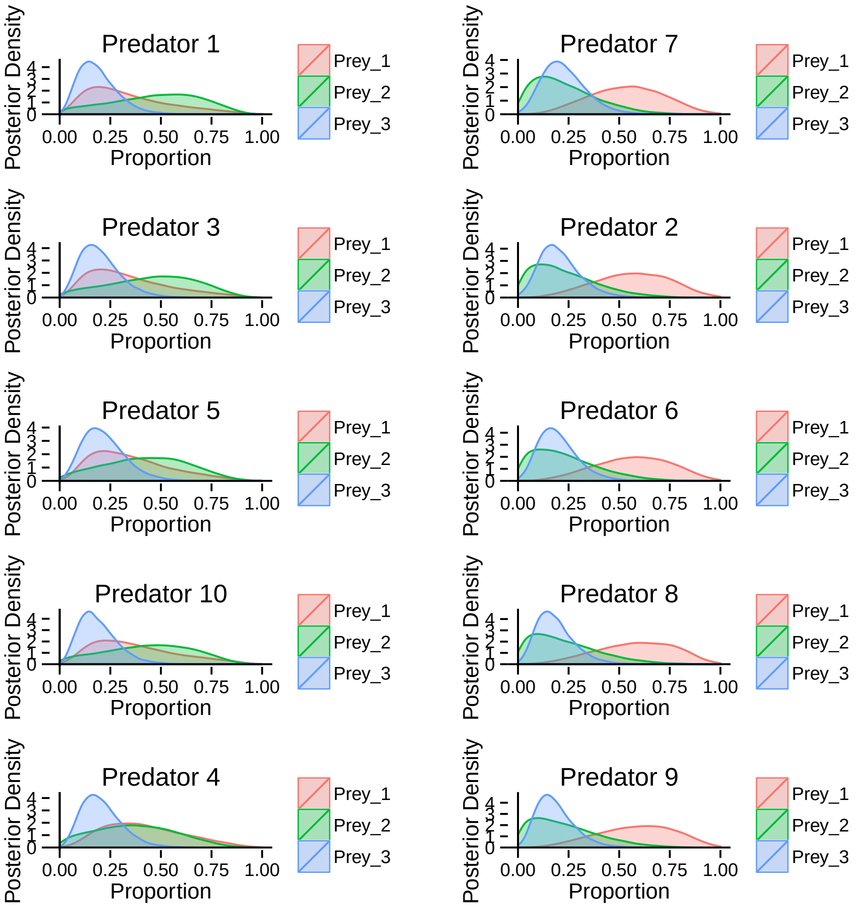

Figure 16: Posterior distributions for individual diet proportions from the FAP analysis

5.2.2 Combined analysis

```
Ind.Combined <- run_MCMC(datas = dats.subset, nIter = 1e+05,
    nBurnin = 10000, nChains = 3, nThin = 100, Data.Type = "Combined.Analysis",
    Analysis.Type = "Individual.proportions", Rnot = 1,
    Rnot_SI = 1, even = 2, plott = F, spawn = T)
```

Again looking at diagnostics output:

```
MCMCplot(Ind.Combined)

diags(Ind.Combined)

summary(Ind.Combined)
```

```
plot(Ind.Combined, save = F, types = "post")

## Using  as id variables
## Using rep(i, nrow(outs)) as id variables
```

The uncertainty for predators preying predominantly on Prey 2 is significantly reduced (but once again the analysis should be run for an order of magnitude longer with a larger thinning interval to ensure that the estimates are reliable).

While the patterns here offer great insights into individual diet proportions, they do not provide a means to estimate the population distribution of diet proportions for these two groups. This can be achieved in an anova type linear model:

5.3 Estimating group proportions

We now need to add covariates in the form of group membership. The reasoning and procedure is the same for continuous covariates (e.g., size). To add the covariates, we use the *add_Covs*constructor:

```
group.path <- system.file("extdata", "Simdata_Groups.csv",
    package = "fastinR")
Covs <- add_Covs(Groups = group.path)

Cov.Combined <- run_MCMC(datas = dats.subset, Covs = Covs,
    nIter = 1e+05, nBurnin = 10000, nChains = 3, nThin = 100,
    Data.Type = "Combined.Analysis", Analysis.Type = "Analysis.with.Covariates",
    Rnot = 1, Rnot_SI = 1, even = 2, plott = F, spawn = T)
```

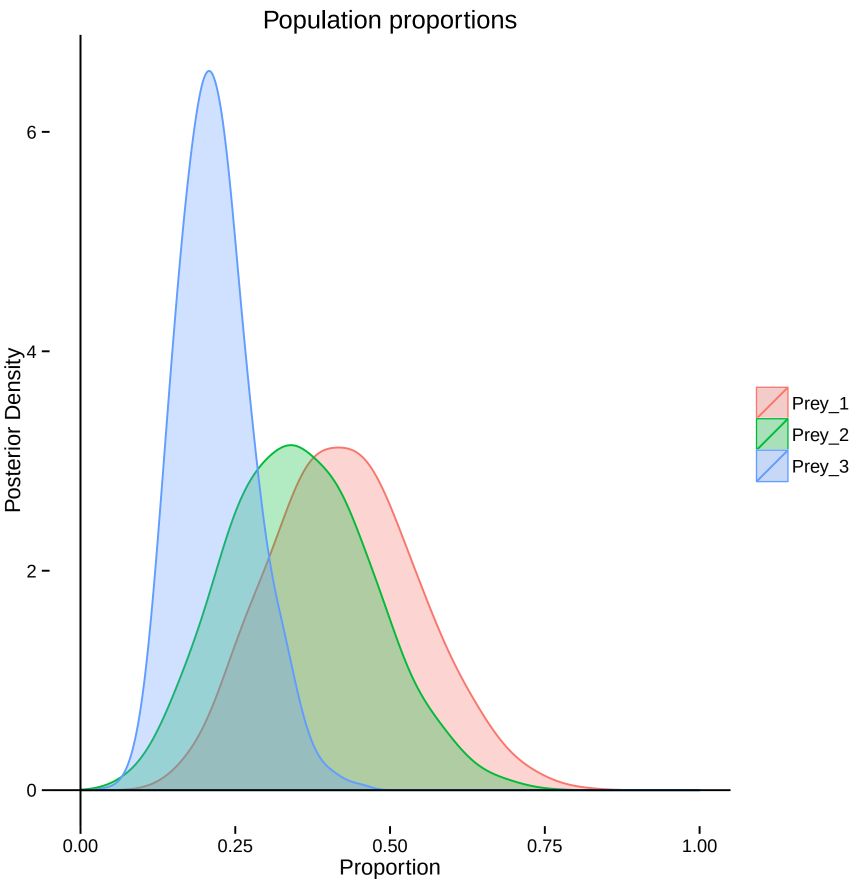

Figure 17: Posterior distributions for individual diet proportions from the combined analysis

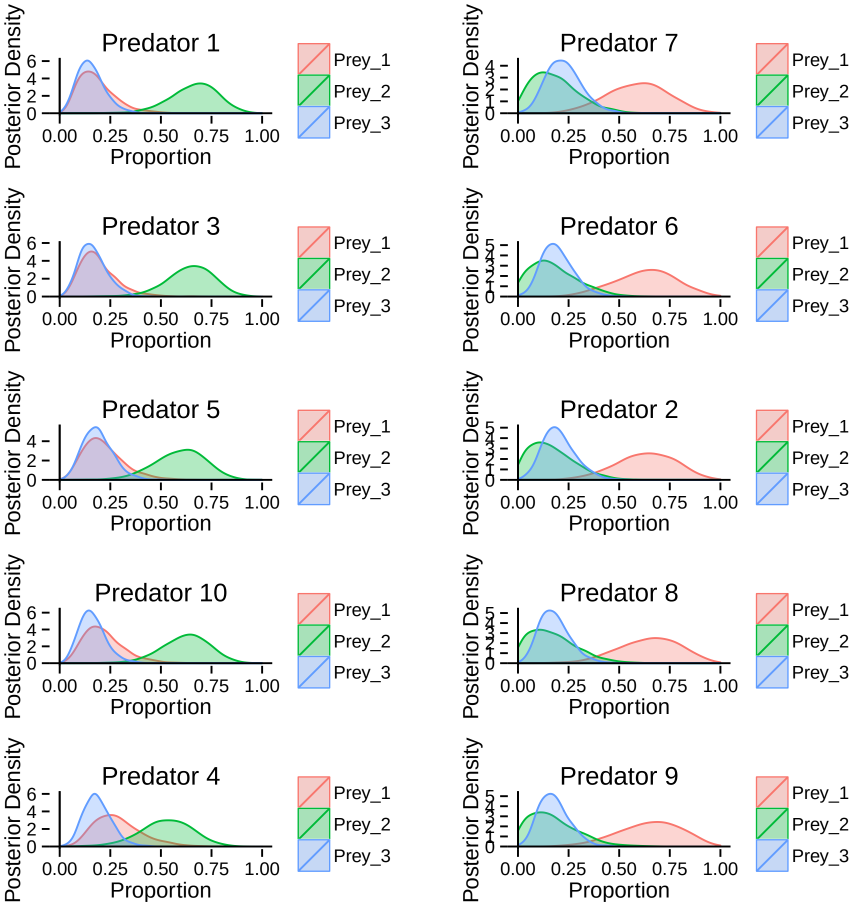

Figure 18: Posterior distributions for individual diet proportions from the combined analysis

```
MCMCplot(Cov.Combined)

diags(Cov.Combined)

summary(Cov.Combined)
```

```
plot(Cov.Combined, save = F, types = "post")

## Using  as id variables
## Using  as id variables
## Using rep(i, nrow(outs)) as id variables
```

We can now clearly see the difference between the two group's diet proportions.

6 Sensitivity illustration

This section illustrates the model sensitivity to source separation and diet evenness. The package has simulated datasets for this illustration, names for their diet evenness (even vs uneven) and source separation (good vs bad). These can be loaded with:

```
data(sensitivity)
```

To visualize, as above,

```
dataplot(even_good)
```

This is an exaggerated example of well separated diets and relatively even diet proportions in predators. Consequently, the model should estimate diet proportions accurately.

A table to compare against plots:

```
rg <- rbind(colMeans(good_prop), apply(good_prop, 2,
    range))
ru <- rbind(colMeans(uneven_prop), apply(uneven_prop,
    2, range))
rb <- rbind(colMeans(bad_prop), apply(bad_prop, 2,
    range))

colnames(rg) <- sprintf("Prey %d", 1:4)
colnames(ru) <- sprintf("Prey %d", 1:4)
colnames(rb) <- sprintf("Prey %d", 1:4)
```

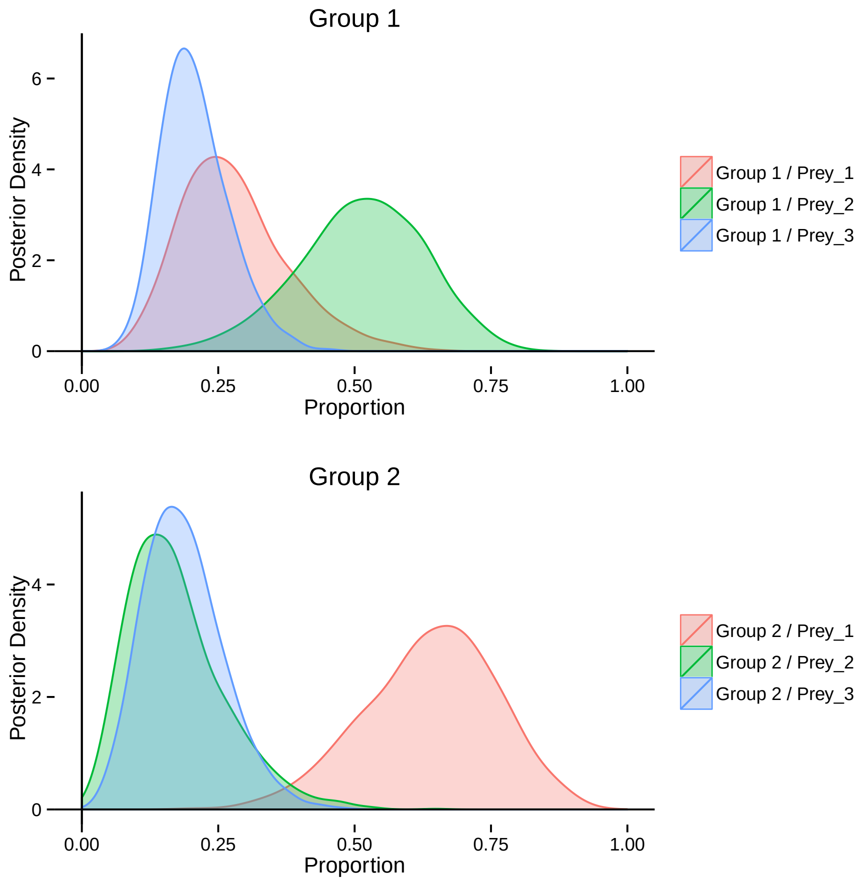

Figure 19: Posterior distributions for individual and group diet proportions from the combined analysis

Table 1: Simulated diet proportions for a simulation with even diet proportions and good resolution of diet items

	Mean π	Min π	Max π
Prey 1	0.19	0.02	0.34
Prey 2	0.27	0.03	0.45
Prey 3	0.16	0.06	0.38
Prey 4	0.39	0.27	0.50

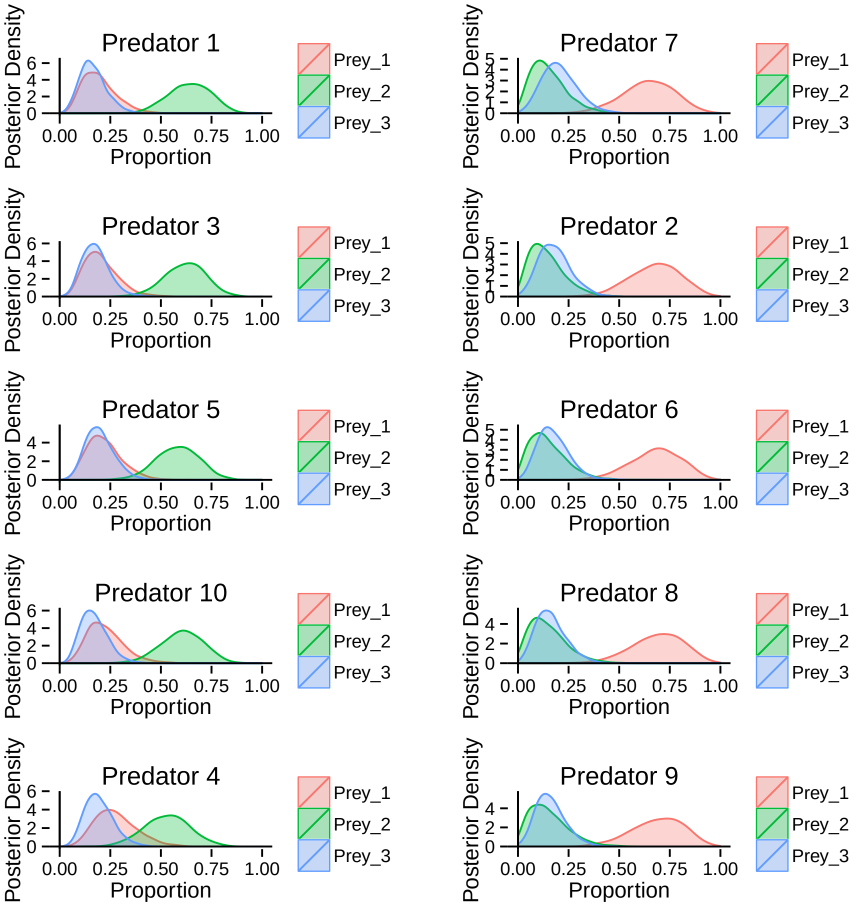

Figure 20: Posterior distributions for individual and group diet proportions from the combined analysis

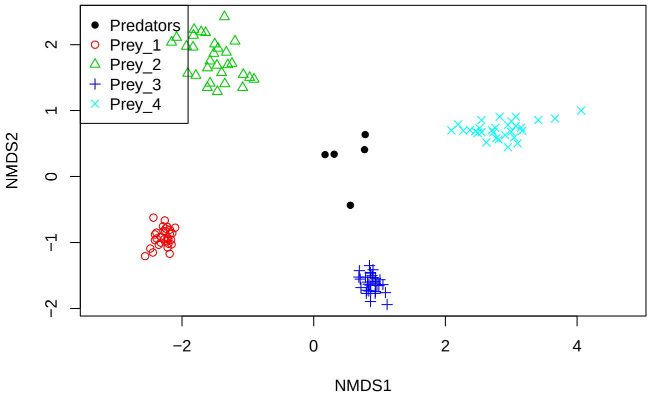

Figure 21: FAP from simulation with even diet proportions and good resolution of diet items projected by NMDS

Table 2: Simulated diet proportions for a simulation with uneven diet proportions and good resolution of diet items

	Mean π	Min π	Max π
Prey 1	0.15	0.03	0.35
Prey 2	0.70	0.39	0.83
Prey 3	0.07	0.01	0.12
Prey 4	0.08	0.00	0.25

```
dataplot(uneven_good)
```

Here, prey separation is good, but diets are skewed towards prey 2.

```
dataplot(even_bad)
```

Lastly, the prey separation is OK for prey species 1, 3 & 4 but prey species 2 has highly variable FA signatures leading to a wide distribution of Prey 2 signatures on the NMDS plot.

For all three examples, we'll use all 10 simulated FAs to make sure that the results are not affected by variable selection. We can run the MCMC directly:

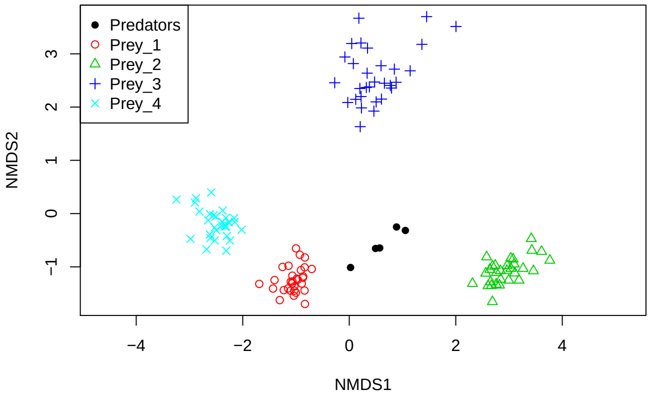

Figure 22: FAP from simulation with uneven diet proportions and good resolution of diet items projected by NMDS

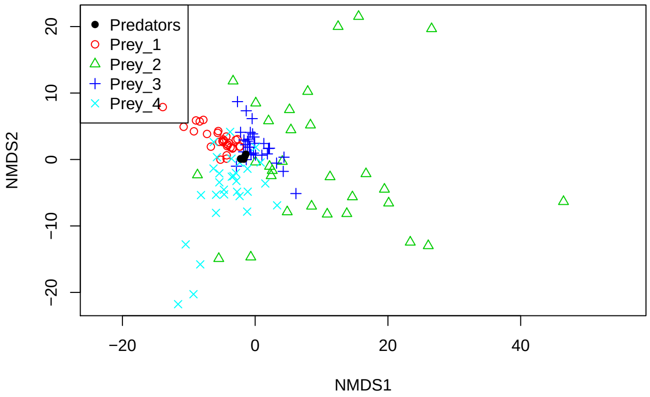

Figure 23: FAP from simulation with somewhat even diet proportions and bad resolution of diet items projected by NMDS

Table 3: Simulated diet proportions for a simulation with somewhat even diet proportions and bad resolution of diet items

	Mean π	Min π	Max π
Prey 1	0.52	0.41	0.67
Prey 2	0.19	0.11	0.31
Prey 3	0.14	0.01	0.33
Prey 4	0.16	0.06	0.21

```r
even.good.mcmc <- run_MCMC(datas = even_good, nIter = 30000,
    nBurnin = 5000, nChains = 3, nThin = 30, Data.Type = "Fatty.Acid.Profiles",
    Analysis.Type = "Population.proportions", Rnot = 2,
    plott = F, spawn = T)



uneven.good.mcmc <- run_MCMC(datas = uneven_good, nIter = 30000,
    nBurnin = 5000, nChains = 3, nThin = 30, Data.Type = "Fatty.Acid.Profiles",
    Analysis.Type = "Population.proportions", Rnot = 2,
    plott = F, spawn = T)



even.bad.mcmc <- run_MCMC(datas = even_bad, nIter = 30000,
    nBurnin = 5000, nChains = 3, nThin = 30, Data.Type = "Fatty.Acid.Profiles",
    Analysis.Type = "Population.proportions", Rnot = 2,
    plott = F, spawn = T)
```

Checking the MCMC:

```r
MCMCplot(even.good.mcmc)
MCMCplot(uneven.good.mcmc)
MCMCplot(even.bad.mcmc)
```

```r
summary(even.good.mcmc)
summary(uneven.good.mcmc)
summary(even.bad.mcmc)
```

The first simulation of idealised data shows that the model clearly recovers the variability and magnitude of population diet proportions. Even for uneven diet proportions, the model performs well, with posteriors reflecting simulated diets. For the last case with low resolution, the posteriors are wide and substantial uncertainty thus reflects the inability to making clear statements about diet proportions.

```r
plot(even.good.mcmc, save = F, types = "post")

## Using  as id variables
```

```r
plot(uneven.good.mcmc, save = F, types = "post")

## Using  as id variables
```

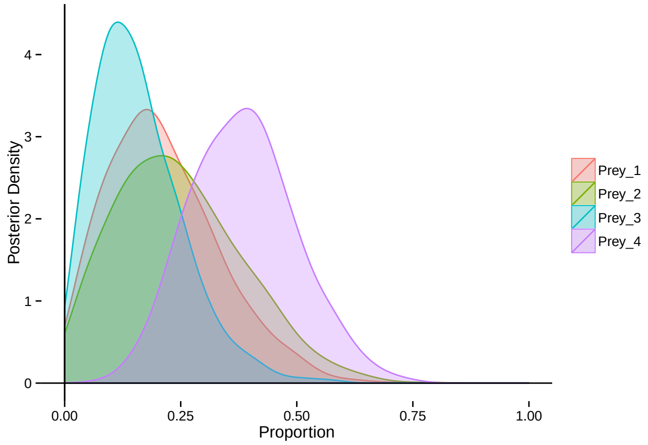

Figure 24: FAP from sensitivity simulations

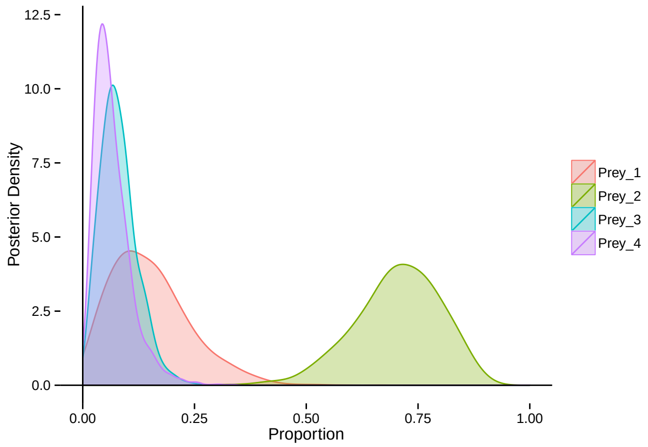

Figure 25: FAP from sensitivity simulations

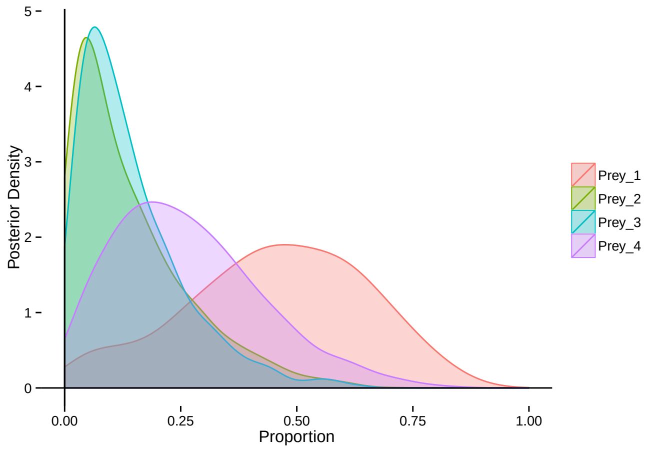

Figure 26: FAP from sensitivity simulations

```
plot(even.bad.mcmc, save = F, types = "post")

## Using  as id variables
```
