# Supplementary material for: Bayesian estimation of predator diet composition from fatty acids and stable isotopes
Source: PeerJ. 2015 Apr 23;3:e920. doi: 10.7717/peerj.920 (PMC4411522; doi:10.7717/peerj.920)
Supplement: Supplemental Information 2 [file peerj-03-920-s002.pdf]

Overall model performance and determinants of estimation accuracy

Philipp Neubauer*

Dragonfly Science,
PO Box 27535, Wellington 6141, New Zealand

Olaf P. Jensen

Institute of Marine and Coastal Sciences
Rutgers University, New Brunswick, NJ 08901, USA

February 2, 2015

1 Simulations to determine factors influencing model performance

1.1 Overall determinants of model performance

Performance of our model can be assessed by measuring the accuracy of estimated diet proportions, which can be tested using simulations. There are a number of potential determinants of accuracy, such as (i) the data used to infer diet proportions, (ii) the model setup as well as (iii) the choice of posterior summary as point estimate of true diet proportions.

At the level of the data input (i), the resolution that the FAPs provide for a given set of potential prey species (i.e., separation of distributions in FAP space), as well as their co-linearity in FAP space will be major determinants of accuracy. Furthermore, the accuracy of conversion coefficients will bear strongly on the model's ability to estimate correct diet proportions (Iverson *et al.*, 2004). In the following, we will illustrate the respective effect of each of these factors using simulations.

*Corresponding author electronic address: neubauer.phil@gmail.com

For the model setup (ii), the model formulation itself (i.e., major assumptions) may more or less suited for the data at hand, leading to variability in accuracy, or specific components of the model may be more or less suited for a particular dataset. The latter case refers to distributional forms of priors and likelihoods formulated in the model, and can be assessed by varying priors and distributional assumptions, whereas the former (e.g., model-mis-specification) is more difficult to measure and should be carefully assessed by the practitioner both *a priori* and by checking the model output.

To illustrate model performance relative to characteristics of the input data (i), we performed a series of 675 standardized simulations that were designed to isolate the relative effect of the evenness of diet proportions, the separation of prey distributions in FAP space, as well as their co-linearity in that space. We further performed simulations to assess the effects of conversion coefficients on diet proportion estimates. As the model setup (ii) and appropriateness of posterior summaries (iii) will depend on both the input data and actual (here, simulated) diet proportions, we standardized the model setup (and priors). For each simulation, we drew 20 samples, containing 12 FAs from each of 4 prey species at random from a Dirichlet distribution with randomly chosen parameters. We then calculated expected predator signatures for 3 predators according to random diet proportions drawn from a random population mean. We then used a model with default priors to estimate population level diet proportions for these predators.

To assess the accuracy in estimated diet proportions, we used posterior means as point estimates of inferred diet proportions for all simulations. We then used a log-linear model to quantify the influence of diet evenness, co-linearity and source separation in FAP space, as well as their interaction, on differences between simulated and inferred diet proportions as measured by the Aitchison distance (Aitchison *et al.*, 2000). We used stepwise model selection using AIC implemented in R (the step function) to select influential determinants of accuracy as well as their interactions.

1.2 Conversion coefficients and their importance for diet estimates

Fatty acids are not always assimilated in direct proportion to their prevalence in a consumers diet. Controlled feeding studies suggest that the relative rate at which individual fatty acids are assimilated may vary by predator, prey, and fatty acid (Rosen & Tollit, 2012). While a detailed discussion of the biochemistry of fatty acid assimilation is beyond the scope of this paper, it is important to note that these coefficients are difficult to obtain from anything but controlled feeding experiments, which in turn are difficult to realize on animals that are not easily cultured or kept in captivity. Many studies that have used FAP to estimate diets have noted potential biases from unknown conversion coefficients (e.g. Iverson *et al.*, 2004; Meynier *et al.*, 2010), and results from experimental studies confirm that the assumption of no and/or false conversion coefficients can bias diet estimates (Rosen & Tollit, 2012). Furthermore, even closely related species may have significantly different conversion coefficients for different fatty acids and prey items (Rosen & Tollit, 2012), and it may therefore be difficult to use a set of coefficients from a closely related predator or prey species for any diet study.

We assessed the effect of ignoring conversion coefficients in our model by estimating simulated diet proportions, first setting κ_s to the true means and variances used to simulate the data, and then running the model with $\kappa_s = 1$, with a prior variance set to the variance κ. To estimate the diet proportions, we used our model on simulated FAPs composed of 12 FAs, simulating prey and FA specific conversion coefficients from a normal distribution centered on 1 and truncated at 0, with increasing variance. This was repeated for the 100 simulated datasets for each of six increments (from 0 to 0.5) in the variance of simulated κ.

2 Simulation results

Simulations illustrated that inferred diets using posterior means were sensitive to separation of diet distributions ($P < 0.05$) and diet evenness ($P < 0.001$), with more even and well separated diets leading to more accurate diet estimates. Co-linearity was not significant in the linear model, likely due to

the simulation setup of only including 4 sources. Blanchard (2011) found a strong influence of source co-linearity with a higher number of sources, and the same should be expected here as a mathematical inevitability.

The interaction between source separation and diet evenness, demonstrated that uneven diets only consistently affect model inferences when sources are not well separated: with increasing separation (Figure 1), the uncertainty about the influence of diet evenness on estimation accuracy increases substantially.

With adequate resolution and known conversion coefficients, diet estimates are usually precise (Figure 2). When conversion coefficients are unknown and set to 1, we found that, on average, the distances between (transformed) diet proportion vectors and their estimates increased with the variance of the conversion coefficients κ. There is no noticeable difference between specifying normal or log-normal models for conversion coefficients. This contrasts with models with specified κ, which have consistently high accuracy, mainly associated with taking point estimates from the posterior distribution to calculate the distance.

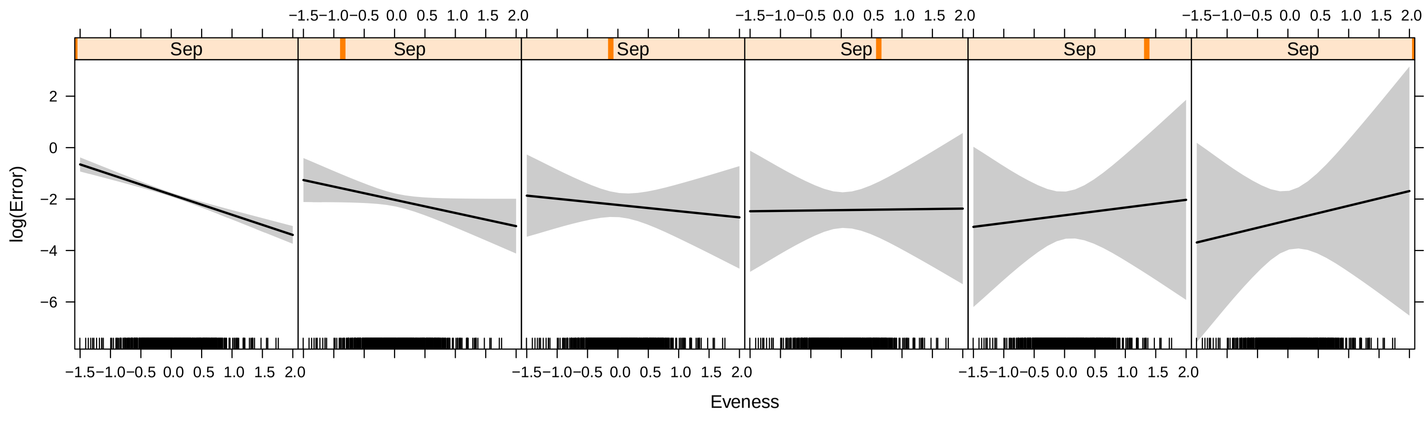

Figure 1: Interactive effect of source separation (Sep) and diet evenness on log distances between simulated diet proportions and posterior means of diet proportions.

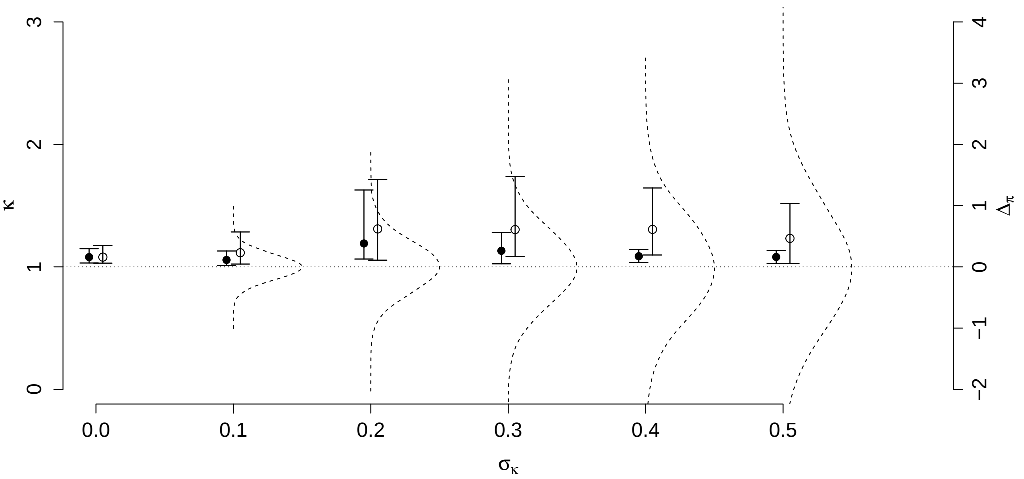

Figure 2: Estimated distance Δ_π between simulated diet proportions and point estimates of diet proportions, as a function on variance of conversion coefficients (σ_κ). Circles are mean distances from point estimates for diet contributions from 100 simulations, error bars are sd of distances. Filled circles show distances from estimates with correct conversion coefficients, open symbols illustrate distances when ignoring these coefficients and instead setting a large variance to reflect uncertainty.

Note that much of the increase in the distance Δ_π between simulated diet proportions and point estimates of diet proportions when ignoring conversion coefficients also results from making point estimates of diet proportions from skewed and wide distributions, reflecting slow or non-convergence of MCMC and/or uncertainty in diet estimates (Figure 3).

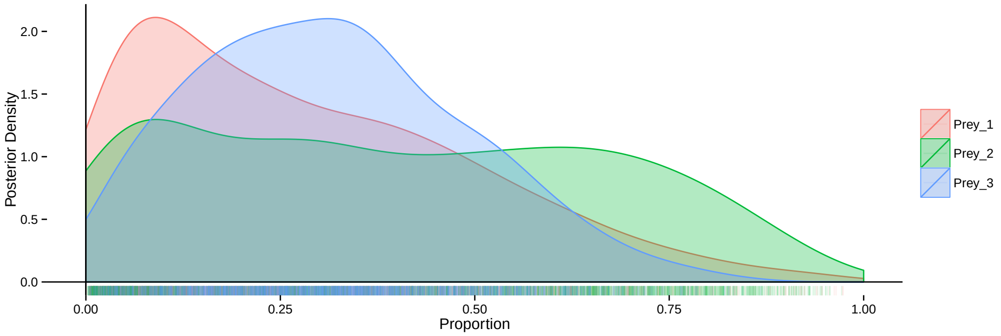

Figure 3: Example of posterior distributions for a simulation with κ set to one, showing the difficulty to obtain reasonable point estimates with ignored conversion coefficients in this case.

References

Aitchison, J., Barcelo-Vidal, C., Martin-Fernandez, J. & Pawlowsky-Glahn, V. (2000). Logratio analysis and compositional distance. *Mathematical Geology*, 32, 271–275.

Blanchard, J. W. (2011). Inference on the diet composition of predators using fatty acid signatures: an application of Bayesian inference on lienar mixing models. PhD thesis. Dalhousie University.

Iverson, S. J., Field, C., Don Bowen, W. & Blanchard, W. (2004). Quantitative fatty acid signature analysis: a new method of estimating predator diets. *Ecological Monographs*, 74, 211–235.

Meynier, L., Morel, P. C., Chilvers, B. L., Mackenzie, D. D. & Duignan, P. J. (2010). Quantitative fatty acid signature analysis on New Zealand sea lions: model sensitivity and diet estimates. *Journal of Mammalogy*, 91, 1484–1495.

Rosen, D. & Tollit, D. (Oct. 2012). Effects of phylogeny and prey type on fatty acid calibration coefficients in three pinniped species: implications for the QFASA dietary quantification technique. *Marine Ecology Progress Series*, 467, 263–276.
