# Supplementary material for: Bayesian estimation of predator diet composition from fatty acids and stable isotopes
Source: PeerJ. 2015 Apr 23;3:e920. doi: 10.7717/peerj.920 (PMC4411522; doi:10.7717/peerj.920)
Supplement: Supplemental Information 3 [file peerj-03-920-s003.pdf]

Selecting fatty acids for diet analysis: an ordination approach

Philipp Neubauer*

Dragonfly Science,
PO Box 27535, Wellington 6141, New Zealand

Olaf P. Jensen

Institute of Marine and Coastal Sciences
Rutgers University, New Brunswick, NJ 08901, USA

February 2, 2015

Given the data transformations applied in our model, choosing a large number of fatty acids (FAs) becomes computationally prohibitive. This is in part due to the fully Bayesian implementation, and an empirical Bayes method could provide more flexibility in the future. Although model complexity scales with the number of FAs, individual predators and prey species, transformation of FAs disproportionately affect computation time. It is thus inevitable to choose an appropriate subset of FAs for diet analysis. When FA profiles are obtained (typically from gas chromatography), the practitioner faces the choice of which FAs out of the potentially large number of measured FAs to retain for the analysis of diet proportions. Typically, only some fatty acids will be informative about diets by separating sources in multivariate space. Adding FAs beyond these informative FAs does not improve estimates of diet proportions, but may instead increase co-linearity among prey samples..

While most studies quantify and list the most abundant FAs, these may not be the most informative to discriminate among potential prey species. Choosing FAs with experimentally validated conversion coefficients is another important consideration. Eliminating FAs with conversion coefficients that are unknown and suspected to be far from 1 is an important first step since their inclusion can introduce significant uncertainty and error in point

*Corresponding author electronic address: neubauer.phil@gmail.com

estimates. Once this preliminary sorting is complete, we propose to select variables based on their contribution to axes in a constrained ordination. We specifically use Constrained Analysis of Principal Coordinates (Anderson & Willis, 2003) since it can deal with any distance metric, and use compositional distance as a distance metric for ordination Aitchison *et al.*, 2000. For each FA f, we sum over the product of the FA contribution to the ordination axes and the axes respective eigenvalues: $\alpha_f = \sum_{a=1}^{n-1} \lambda_a c_{f,a}$, where a indexes individual ordination axes, and $c_{f,a}$ is the contribution of FA f to axis a. Each α_f then contributes a proportion p to $A = \sum_f \alpha_f$, and we can order the α_f by their contribution to A and choose a number of variables that contribute to a cumulative proportion P of the cumulative separation A.

References

Aitchison, J., Barcelo-Vidal, C., Martin-Fernandez, J. & Pawlowsky-Glahn, V. (2000). Logratio analysis and compositional distance. *Mathematical Geology*, 32, 271–275.

Anderson, M. J. & Willis, T. J. (2003). Canonical analysis of principal coordinates: a useful method of constrained ordination for ecology. *Ecology*, 84, 511–525.
