# Supplementary material for: Bayesian estimation of predator diet composition from fatty acids and stable isotopes
Source: PeerJ. 2015 Apr 23;3:e920. doi: 10.7717/peerj.920 (PMC4411522; doi:10.7717/peerj.920)
Supplement: Supplemental Information 4 [file peerj-03-920-s004.pdf]

Discrimination Analysis

Philipp Neubauer

Dragonfly Science,
PO Box 27535, Wellington 6141, New Zealand

June 11, 2014

Preamble

This document details the estimation procedure for stable isotope discrimination and fatty acid conversion coefficients. The data used is from Stowasser et al 2006 (JEMBE 333: 97–114), and is available as .csv files in the same folder as this document on github. We group all fish together as we don't know the proportions of individual fish species that were fed in the treatment. The uncertainty in estimated discrimination coefficients will reflect any variability in species specific coefficients, so will mainly just introduce additional uncertainty in the final analysis.

NOTE: The fatty acid analysis takes a long time to run (possibly several hours, depending on CPU speed and number of cores). Please factor this in when considering to repeat/modify this analysis.

Stable Isotope discrimination

Start with estimating SI discrimination based on fish and crustacean only diets. First we read the .csv files with raw data:

Data grooming

```
library(knitr)
options(replace.assign = TRUE, width = 50)

prey.table <- read.csv("Prey_SI.csv", header = T)
pred.table <- read.csv("Predator_SI.csv", header = T, stringsAsFactors = F)
```

We'll need a few items later, such as the number of preys, here set to two - fish and crustacean - and an index of prey samples.

```
# index
prey.type <- as.numeric(prey.table[, 1] == "Grass shrimp") +
    1
# number of prey types
n.preys <- 2
# total number of prey samples
n.preys.samps <- length(prey.type)
# final prey table
prey <- prey.table[, 2:3]
```

Similarly, for the predators we'll need a feed index to identify which food each predator was fed on.

```
# feed type index
feed.type <- pred.table[, 1]
# fish
feed.type[pred.table[, 1] == "F"] <- 1
# crustacean
feed.type[pred.table[, 1] == "C"] <- 2
# use only fish and crustacean diets, rest will be
# used for diet estimation subset
idx <- which(feed.type %in% c(1, 2))
feed.type <- as.numeric(feed.type[idx])

# subset predator table in the same way
pred <- pred.table[idx, 2:3]
# number of predator samples
n.preds.samps <- length(idx)
```

Priors

We now set up priors for the analysis. We need priors for both the intercept and concentration dependence of the priors. These are most parsimoniously set from meta-analyses: Priors for ΔN follow from Hussey et al 2014 (Ecology Letters Volume 17, Issue 2, pages 239250, February 2014). Priors for ΔC are from Caut et al 2009 (Journal of Applied Ecology, 46, 443453).

```
# beta.not - 95% interval contains 2*2SD, if
# symmetric then
bnot.prior.N <- 5.92
sd.bnot.N <- (5.92 - 4.55)/2
bnot.tau.prior.N <- 1/(sd.bnot.N^2)

beta.prior.N <- -0.27
```

```
sd.beta.N <- (-0.27 - -0.41)/2
beta.tau.prior.N <- 1/(sd.beta.N^2)

# no variance given in Caut, set prior to clearly
# larger than in N
bnot.prior.C <- -2.85
sd.bnot.C <- 2
bnot.tau.prior.C <- 1/(sd.bnot.C^2)

beta.prior.C <- -0.21
sd.beta.C <- 0.4
beta.tau.prior.C <- 1/(sd.beta.C^2)

# final priors
bnot.prior <- c(bnot.prior.C, bnot.prior.N)
bnot.tau.prior <- c(bnot.tau.prior.C, bnot.tau.prior.N)
beta.prior <- c(beta.prior.C, beta.prior.N)
beta.tau.prior <- c(beta.tau.prior.C, beta.tau.prior.N)
```

We simply set the prior mean for the prey mean to the sample mean, but leave the variance vague so as to not force the mean.

```
require(dplyr)
# predator mean priors
prior.mu <- data.frame(feed = prey.type, prey) %.%
    group_by(feed) %.% summarise(mu.C = mean(X_13C),
    mu.N = mean(X_15N)) %.% arrange(feed)

prior.mu <- as.matrix(prior.mu[, 2:3])
```

Jags analysis

This is all put into the appropriate format for jags, and off we go...

```
input <- list(prey = prey, pred = pred, n.preys.samps = n.preys.samps,
    n.preys = n.preys, n.preds.samps = n.preds.samps,
    prey.type = prey.type, feed.type = feed.type, prior.mu = prior.mu,
    bnot.prior = bnot.prior, beta.prior = beta.prior,
    bnot.tau.prior = bnot.tau.prior, beta.tau.prior = beta.tau.prior)

require(rjags)

## Loading required package:  rjags
## Loading required package:  coda
## Loading required package:  lattice
```

```
## Linked to JAGS 3.4.0
## Loaded modules:  basemod,bugs

DM <- jags.model("Discrim.model.SI.R", n.chains = 3,
    inits = list(mu = prior.mu, beta.not = bnot.prior,
        beta.reg = beta.prior), data = input)
```

```
update(DM, 10000)

samps <- coda.samples(DM, c("pred.discr", "beta.reg",
    "beta.not", "mu"), n.iter = 1e+05, thin = 100)
```

All done, checking output visually for adequacy (convergence, lack of strong autocorrelation in the Markov Chains) , extract estimated coefficients for each preys species and isotope and put the resulting matrix into the right format.

```
plot(samps, ask = T)  # need to run this outside of compilation
```

```
summary(samps)

##
## Iterations = 10100:110000
## Thinning interval = 100
## Number of chains = 3
## Sample size per chain = 1000
##
## 1. Empirical mean and standard deviation for each variable,
##    plus standard error of the mean:
##
##                    Mean     SD Naive SE
## beta.not[1]      -2.481 1.9264  0.03517
## beta.not[2]       7.602 0.4708  0.00860
## beta.reg[1]      -0.233 0.1139  0.00208
## beta.reg[2]      -0.181 0.0674  0.00123
## mu[1,1]         -20.025 0.4940  0.00902
## mu[2,1]         -17.126 0.3774  0.00689
## mu[1,2]           5.721 0.3893  0.00711
## mu[2,2]           4.316 0.2674  0.00488
```

```
## pred.discr[1,1]   2.176 0.5648  0.01031
## pred.discr[2,1]   1.509 0.4517  0.00825
## pred.discr[1,2]   6.567 0.4979  0.00909
## pred.discr[2,2]   6.818 0.4841  0.00884
##                Time-series SE
## beta.not[1]           0.04471
## beta.not[2]           0.00836
## beta.reg[1]           0.00267
## beta.reg[2]           0.00123
## mu[1,1]               0.00903
## mu[2,1]               0.00689
## mu[1,2]               0.00668
## mu[2,2]               0.00468
## pred.discr[1,1]       0.01153
## pred.discr[2,1]       0.00824
## pred.discr[1,2]       0.00909
## pred.discr[2,2]       0.00886
##
## 2. Quantiles for each variable:
##
##                    2.5%     25%     50%     75%
## beta.not[1]      -6.160  -3.784  -2.468  -1.217
## beta.not[2]       6.595   7.311   7.609   7.924
## beta.reg[1]      -0.451  -0.311  -0.232  -0.161
## beta.reg[2]      -0.311  -0.227  -0.182  -0.136
## mu[1,1]         -21.004 -20.327 -20.017 -19.709
## mu[2,1]         -17.798 -17.344 -17.143 -16.937
## mu[1,2]           5.067   5.470   5.685   5.939
## mu[2,2]           3.876   4.169   4.295   4.433
## pred.discr[1,1]   1.043   1.835   2.193   2.540
## pred.discr[2,1]   0.603   1.258   1.534   1.785
## pred.discr[1,2]   5.498   6.259   6.602   6.901
## pred.discr[2,2]   5.794   6.528   6.853   7.149
##                   97.5%
## beta.not[1]      1.4719
## beta.not[2]      8.4543
## beta.reg[1]     -0.0022
## beta.reg[2]     -0.0470
## mu[1,1]        -19.0593
## mu[2,1]        -16.3218
## mu[1,2]          6.5409
## mu[2,2]          4.8757
## pred.discr[1,1]  3.2380
## pred.discr[2,1]  2.3723
## pred.discr[1,2]  7.4361
```

```
## pred.discr[2,2]   7.6498
```

It seems that despite the informative priors, the posterior for both beta.not[2] and beta.reg[2] (coeffs for ΔN) are higher and lower, respectively, in their posterior median than their respective priors. The high intercept leads to ΔN discrimination coefficients that are both high and similar in magnitude for both prey species, indicating that this may be linked to the tissue type or to the predator. For ΔC the posterior medians are close to the respective prior means. Estimated ΔC discrimination is positive for both species.

```
# get estimated discrimination from all chains:
ix <- grep("pred.discr", colnames(samps[[1]]))

# combine chains
r.samps <- do.call("rbind", samps)[, ix]
dim(r.samps)

## [1] 3000    4

# into format for analysis
discr.means <- matrix(apply(r.samps, 2, mean), 2, 2)
# pretend discrimination is the same for all fish
# species
discr.means <- rbind(discr.means[1, ], discr.means[1,
    ], discr.means)

discr.var <- matrix(apply(r.samps, 2, var), 2, 2)
# pretend discrimination is the same for all fish
# species
discr.var <- rbind(discr.var[1, ], discr.var[1, ],
    discr.var)

# write to file
colnames(discr.means) <- colnames(prey.table)[2:3]
rownames(discr.means) <- unique(prey.table[, 1])
write.csv(discr.means, file = "discr.means.csv")
discr.means

##                      X_13C X_15N
## Silverside           2.176 6.567
## Sailfin Molly        2.176 6.567
## Sheepshead minnow    2.176 6.567
## Grass shrimp         1.509 6.818

# write to file
colnames(discr.var) <- colnames(prey.table)[2:3]
```

```r
rownames(discr.var) <- unique(prey.table[, 1])
write.csv(discr.var, file = "discr.var.csv")
discr.var

##                    X_13C  X_15N
## Silverside         0.319 0.2479
## Sailfin Molly      0.319 0.2479
## Sheepshead minnow 0.319 0.2479
## Grass shrimp       0.204 0.2343
```

All done for SI, moving on to FAP...

Fatty Acid Conversion Coefficients

Again, we start with data grooming, reading in raw data and preparing it for analysis:

Data grooming

```r
require(fastinR)

## Loading required package:  fastinR
## Warning:  no DISPLAY variable so Tk is not available
##
## Attaching package:  'fastinR'
##
## The following object is masked from 'package:dplyr':
##
##    select_vars
##
## The following object is masked from 'package:utils':
##
##    adist

prey.table.FA <- t(read.csv("Prey_FA.csv", header = F,
    row.names = 1))
pred.table.FA <- t(read.csv("Predator_FA.csv", header = T,
    stringsAsFactors = F, row.names = 1))

# prey index
prey.type <- rep(1, nrow(prey.table.FA))
prey.type[prey.table.FA[, 1] == "Grass Shrimp"] <- 2

# msc
```

```r
n.preys <- 2
n.preys.samps <- length(prey.type)
n.fats <- 25

# replace zeros in compositions with min recorded
# for that SI. This is somewhat arbitrary.
prey.ix <- prey.table.FA[, 1]
prey.table.FA <- matrix(as.numeric(prey.table.FA[,
    2:26]), n.preys.samps, n.fats)
for (i in 1:ncol(prey.table.FA)) {
    prey.table.FA[prey.table.FA[, i] == 0, i] <- min(prey.table.FA[prey.table.FA[,
        i] > 0, i])
}
# log ratio transform
prey <- alr(prey.table.FA)

for (i in 1:ncol(pred.table.FA)) {
    pred.table.FA[pred.table.FA[, i] == 0, i] <- min(pred.table.FA[pred.table.FA[,
        i] > 0, i])
}
# log ratio transform
pred <- alr(pred.table.FA)

# feed type index
feed.type <- pred.table.FA[, 1]
FAs <- colnames(pred.table.FA)
# use only samples that were fed on single diet for
# >=20days
feed.type[which(regexpr("F", rownames(pred.table.FA)) ==
    1)[9:15]] <- 1
# take out an early sample
feed.type[which(regexpr("C", rownames(pred.table.FA)) ==
    1)[c(6:8, 10:13)]] <- 2
# use only fish and crustaceans diets, rest will be
# assessed
idx <- which(feed.type %in% c(1, 2))
feed.type <- as.integer(feed.type[idx])

# final predator table
pred <- pred[idx, ]
n.preds.samps <- length(idx)
```

FAP priors

There seems to be little evidence about consistent patterns in conversion coefficients for FAP, so we won't set any informative priors.

```
# predator mean priors
pt <- aggregate(prey.table.FA, list(prey.type), gmean)
prior.mu <- data.matrix(alr(pt[, 2:length(pt)]))

n.fats <- ncol(prey.table.FA)
m.fats <- n.fats - 1
# reasonably vague priors
S <- diag(0.01, m.fats)
R <- diag(0.01, n.fats)

# uniform prior for Dirichlet on conversion coeffs
p <- rep(1/n.fats, n.fats)
zeros <- rep(0, n.fats)
```

Ready to run JAGS...

```
require(rjags)

DM <- jags.model("Discrim.model.FA.R", n.chains = 3)

## Compiling model graph
##    Resolving undeclared variables
##    Allocating nodes
##    Graph Size: 1203
##
## Initializing model

update(DM, 10000)

samps.FA <- coda.samples(DM, c("beta.reg"), n.iter = 1e+05,
    thin = 100)
```

Again, we examine the output for auto-correlation and convergence. Seems OK, despite the 'long tails'.

```
plot(samps.FA, ask = T)
crosscorr.plot(samps.FA)
```

```
# display posterior summary
summary(samps.FA)
```

```
##
## Iterations = 11100:111000
## Thinning interval = 100
## Number of chains = 3
## Sample size per chain = 1000
##
## 1. Empirical mean and standard deviation for each variable,
##    plus standard error of the mean:
##
##                   Mean      SD Naive SE
## beta.reg[1,1]  0.01778 0.00444 8.11e-05
## beta.reg[2,1]  0.01452 0.00322 5.88e-05
## beta.reg[1,2]  0.02296 0.00479 8.74e-05
## beta.reg[2,2]  0.02114 0.00444 8.10e-05
## beta.reg[1,3]  0.02349 0.00485 8.85e-05
## beta.reg[2,3]  0.02483 0.00435 7.94e-05
## beta.reg[1,4]  0.04231 0.00974 1.78e-04
## beta.reg[2,4]  0.03458 0.00728 1.33e-04
## beta.reg[1,5]  0.01981 0.00447 8.17e-05
## beta.reg[2,5]  0.01262 0.00229 4.18e-05
## beta.reg[1,6]  0.02927 0.00923 1.68e-04
## beta.reg[2,6]  0.00917 0.00424 7.73e-05
## beta.reg[1,7]  0.03603 0.01751 3.20e-04
## beta.reg[2,7]  0.02093 0.01584 2.89e-04
## beta.reg[1,8]  0.04302 0.01758 3.21e-04
## beta.reg[2,8]  0.03410 0.01522 2.78e-04
## beta.reg[1,9]  0.06546 0.01179 2.15e-04
## beta.reg[2,9]  0.06443 0.01352 2.47e-04
## beta.reg[1,10] 0.02002 0.00520 9.50e-05
## beta.reg[2,10] 0.01570 0.00372 6.79e-05
## beta.reg[1,11] 0.02827 0.00830 1.52e-04
## beta.reg[2,11] 0.01711 0.00491 8.96e-05
## beta.reg[1,12] 0.01836 0.00591 1.08e-04
## beta.reg[2,12] 0.01483 0.00288 5.26e-05
## beta.reg[1,13] 0.01757 0.00796 1.45e-04
## beta.reg[2,13] 0.01376 0.00550 1.00e-04
## beta.reg[1,14] 0.01268 0.00374 6.82e-05
## beta.reg[2,14] 0.00917 0.00161 2.93e-05
## beta.reg[1,15] 0.01231 0.00431 7.88e-05
## beta.reg[2,15] 0.01064 0.00363 6.63e-05
## beta.reg[1,16] 0.13930 0.02684 4.90e-04
## beta.reg[2,16] 0.10926 0.01914 3.49e-04
## beta.reg[1,17] 0.03430 0.01124 2.05e-04
## beta.reg[2,17] 0.07260 0.02906 5.30e-04
## beta.reg[1,18] 0.11836 0.02796 5.10e-04
```

```
## beta.reg[2,18] 0.07390 0.01349 2.46e-04
## beta.reg[1,19] 0.02109 0.00487 8.90e-05
## beta.reg[2,19] 0.02740 0.00685 1.25e-04
## beta.reg[1,20] 0.11041 0.02204 4.02e-04
## beta.reg[2,20] 0.06521 0.01209 2.21e-04
## beta.reg[1,21] 0.02928 0.00678 1.24e-04
## beta.reg[2,21] 0.03619 0.01033 1.89e-04
## beta.reg[1,22] 0.03115 0.01230 2.25e-04
## beta.reg[2,22] 0.10811 0.02111 3.85e-04
## beta.reg[1,23] 0.02943 0.00620 1.13e-04
## beta.reg[2,23] 0.07188 0.01273 2.32e-04
## beta.reg[1,24] 0.05328 0.00985 1.80e-04
## beta.reg[2,24] 0.06998 0.01335 2.44e-04
## beta.reg[1,25] 0.02404 0.00696 1.27e-04
## beta.reg[2,25] 0.04793 0.00736 1.34e-04
##                Time-series SE
## beta.reg[1,1]        3.10e-04
## beta.reg[2,1]        2.30e-04
## beta.reg[1,2]        3.49e-04
## beta.reg[2,2]        2.86e-04
## beta.reg[1,3]        2.88e-04
## beta.reg[2,3]        2.22e-04
## beta.reg[1,4]        1.18e-03
## beta.reg[2,4]        3.94e-04
## beta.reg[1,5]        2.68e-04
## beta.reg[2,5]        1.06e-04
## beta.reg[1,6]        1.03e-03
## beta.reg[2,6]        5.14e-04
## beta.reg[1,7]        2.68e-03
## beta.reg[2,7]        2.11e-03
## beta.reg[1,8]        2.08e-03
## beta.reg[2,8]        1.10e-03
## beta.reg[1,9]        6.30e-04
## beta.reg[2,9]        1.30e-03
## beta.reg[1,10]       3.75e-04
## beta.reg[2,10]       2.69e-04
## beta.reg[1,11]       1.06e-03
## beta.reg[2,11]       4.98e-04
## beta.reg[1,12]       5.37e-04
## beta.reg[2,12]       1.67e-04
## beta.reg[1,13]       8.55e-04
## beta.reg[2,13]       6.54e-04
## beta.reg[1,14]       3.24e-04
## beta.reg[2,14]       7.79e-05
## beta.reg[1,15]       3.08e-04
```

```
## beta.reg[2,15]       3.05e-04
## beta.reg[1,16]       1.72e-03
## beta.reg[2,16]       1.00e-03
## beta.reg[1,17]       1.59e-03
## beta.reg[2,17]       2.48e-03
## beta.reg[1,18]       3.28e-03
## beta.reg[2,18]       6.42e-04
## beta.reg[1,19]       3.72e-04
## beta.reg[2,19]       5.02e-04
## beta.reg[1,20]       1.54e-03
## beta.reg[2,20]       6.59e-04
## beta.reg[1,21]       4.78e-04
## beta.reg[2,21]       7.12e-04
## beta.reg[1,22]       9.85e-04
## beta.reg[2,22]       1.25e-03
## beta.reg[1,23]       3.43e-04
## beta.reg[2,23]       7.43e-04
## beta.reg[1,24]       5.96e-04
## beta.reg[2,24]       6.91e-04
## beta.reg[1,25]       1.24e-03
## beta.reg[2,25]       6.13e-04
##
## 2. Quantiles for each variable:
##
##                    2.5%     25%     50%     75%
## beta.reg[1,1]  0.00991 0.01520 0.01738 0.01999
## beta.reg[2,1]  0.00867 0.01266 0.01431 0.01609
## beta.reg[1,2]  0.01362 0.02024 0.02269 0.02543
## beta.reg[2,2]  0.01328 0.01893 0.02092 0.02293
## beta.reg[1,3]  0.01345 0.02075 0.02333 0.02617
## beta.reg[2,3]  0.01525 0.02265 0.02478 0.02694
## beta.reg[1,4]  0.02828 0.03752 0.04085 0.04454
## beta.reg[2,4]  0.02175 0.03097 0.03392 0.03740
## beta.reg[1,5]  0.01125 0.01714 0.01953 0.02215
## beta.reg[2,5]  0.00833 0.01138 0.01245 0.01365
## beta.reg[1,6]  0.01351 0.02330 0.02860 0.03424
## beta.reg[2,6]  0.00416 0.00644 0.00822 0.01068
## beta.reg[1,7]  0.01208 0.02346 0.03244 0.04445
## beta.reg[2,7]  0.00454 0.01026 0.01666 0.02640
## beta.reg[1,8]  0.01879 0.03078 0.03943 0.05139
## beta.reg[2,8]  0.01104 0.02262 0.03169 0.04314
## beta.reg[1,9]  0.03980 0.05960 0.06547 0.07113
## beta.reg[2,9]  0.04177 0.05840 0.06360 0.06933
## beta.reg[1,10] 0.01066 0.01693 0.01978 0.02251
## beta.reg[2,10] 0.01007 0.01392 0.01523 0.01671
```

```
## beta.reg[1,11] 0.01608 0.02434 0.02735 0.03053
## beta.reg[2,11] 0.01069 0.01497 0.01646 0.01825
## beta.reg[1,12] 0.00894 0.01478 0.01783 0.02108
## beta.reg[2,12] 0.00959 0.01333 0.01464 0.01605
## beta.reg[1,13] 0.00719 0.01204 0.01581 0.02122
## beta.reg[2,13] 0.00769 0.01076 0.01275 0.01515
## beta.reg[1,14] 0.00595 0.01028 0.01238 0.01471
## beta.reg[2,14] 0.00613 0.00827 0.00911 0.00993
## beta.reg[1,15] 0.00567 0.00932 0.01177 0.01443
## beta.reg[2,15] 0.00507 0.00834 0.01009 0.01228
## beta.reg[1,16] 0.09146 0.12415 0.13803 0.15246
## beta.reg[2,16] 0.06502 0.10022 0.10938 0.11881
## beta.reg[1,17] 0.01922 0.02872 0.03281 0.03711
## beta.reg[2,17] 0.02545 0.05324 0.06902 0.08794
## beta.reg[1,18] 0.07028 0.10552 0.11569 0.12710
## beta.reg[2,18] 0.04767 0.06659 0.07378 0.08116
## beta.reg[1,19] 0.01325 0.01853 0.02063 0.02289
## beta.reg[2,19] 0.01725 0.02355 0.02657 0.03007
## beta.reg[1,20] 0.06726 0.09744 0.10955 0.12279
## beta.reg[2,20] 0.03954 0.05968 0.06511 0.07070
## beta.reg[1,21] 0.01623 0.02480 0.02903 0.03342
## beta.reg[2,21] 0.01810 0.02923 0.03516 0.04233
## beta.reg[1,22] 0.01281 0.02248 0.02911 0.03751
## beta.reg[2,22] 0.06883 0.09499 0.10642 0.11928
## beta.reg[1,23] 0.01805 0.02575 0.02912 0.03291
## beta.reg[2,23] 0.04546 0.06530 0.07156 0.07827
## beta.reg[1,24] 0.03165 0.04863 0.05345 0.05823
## beta.reg[2,24] 0.04546 0.06313 0.06947 0.07587
## beta.reg[1,25] 0.01379 0.01869 0.02300 0.02843
## beta.reg[2,25] 0.03532 0.04287 0.04692 0.05242
##                  97.5%
## beta.reg[1,1]  0.0272
## beta.reg[2,1]  0.0220
## beta.reg[1,2]  0.0335
## beta.reg[2,2]  0.0306
## beta.reg[1,3]  0.0339
## beta.reg[2,3]  0.0346
## beta.reg[1,4]  0.0745
## beta.reg[2,4]  0.0504
## beta.reg[1,5]  0.0300
## beta.reg[2,5]  0.0183
## beta.reg[1,6]  0.0510
## beta.reg[2,6]  0.0209
## beta.reg[1,7]  0.0840
## beta.reg[2,7]  0.0713
```

```
## beta.reg[1,8]  0.0887
## beta.reg[2,8]  0.0690
## beta.reg[1,9]  0.0901
## beta.reg[2,9]  0.0940
## beta.reg[1,10] 0.0312
## beta.reg[2,10] 0.0241
## beta.reg[1,11] 0.0468
## beta.reg[2,11] 0.0274
## beta.reg[1,12] 0.0312
## beta.reg[2,12] 0.0212
## beta.reg[1,13] 0.0377
## beta.reg[2,13] 0.0275
## beta.reg[1,14] 0.0210
## beta.reg[2,14] 0.0128
## beta.reg[1,15] 0.0230
## beta.reg[2,15] 0.0195
## beta.reg[1,16] 0.1949
## beta.reg[2,16] 0.1498
## beta.reg[1,17] 0.0642
## beta.reg[2,17] 0.1393
## beta.reg[1,18] 0.1845
## beta.reg[2,18] 0.1008
## beta.reg[1,19] 0.0317
## beta.reg[2,19] 0.0437
## beta.reg[1,20] 0.1580
## beta.reg[2,20] 0.0906
## beta.reg[1,21] 0.0430
## beta.reg[2,21] 0.0581
## beta.reg[1,22] 0.0618
## beta.reg[2,22] 0.1566
## beta.reg[1,23] 0.0420
## beta.reg[2,23] 0.1004
## beta.reg[1,24] 0.0742
## beta.reg[2,24] 0.0987
## beta.reg[1,25] 0.0406
## beta.reg[2,25] 0.0648
```

And process the output for the diet analysis.

```
# get estimated discrimination from all chains:
r.samps <- do.call("rbind", samps.FA)
dim(r.samps)

## [1] 3000   50

fish.cc.samples <- r.samps[, seq(1, 2 * n.fats, 2)]
```

```r
shrimp.cc.samples <- r.samps[, seq(2, 2 * n.fats, 2)]

fish.cc <- colMeans(fish.cc.samples)
shrimp.cc <- colMeans(shrimp.cc.samples)

# combine and write to file, repeat for 3 fish
# species in final analysis
ccs <- rbind(fish.cc, fish.cc, fish.cc, shrimp.cc)
rownames(ccs) <- unique(prey.ix)[-4]
colnames(ccs) <- colnames(pred.table.FA)
write.csv(ccs, file = "cc_FA.csv")

# using independent ccs seems warranted here given
# cross-corr plot
fish.cc.var <- apply(fish.cc.samples, 2, var)
shrimp.cc.var <- apply(shrimp.cc.samples, 2, var)
# combine
ccs.var <- rbind(fish.cc.var, fish.cc.var, fish.cc.var,
    shrimp.cc.var)
rownames(ccs.var) <- unique(prey.ix)[-4]
colnames(ccs.var) <- colnames(pred.table.FA)
write.csv(ccs.var, file = "cc_FA_var.csv")
```

All done.
