# Supplementary material for: Bayesian estimation of predator diet composition from fatty acids and stable isotopes
Source: PeerJ. 2015 Apr 23;3:e920. doi: 10.7717/peerj.920 (PMC4411522; doi:10.7717/peerj.920)
Supplement: Supplemental Information 5 [file peerj-03-920-s005.pdf]

Analysis of Stowasser et al. 2006 experimental data

Philipp Neubauer

Dragonfly Science,
PO Box 27535, Wellington 6141, New Zealand

June 11, 2014

Preamble

Here we demonstrate our methods by re-analysing the experimental dataset of Stowasser et al. 2006 (JEMBE 333: 97–114). The original paper investigated the evolution of stable isotopes (SI, $\delta^{15}C$ and $\delta^{15}N$) and fatty acid (FA) signatures for squid fed on fish, crustacean or mixed diets, as well as switched diet regimes. Our specific aim here was to estimate diet proportions of switched diet treatment individuals, and to show the advantage of using both markers in concert over using a single marker for diet analysis.

We begin the analysis using just SI. Hussey et al 2014 (Ecology Letters Volume 17, Issue 2, pages 239–250, February 2014), following earlier analyses by Caut et al 2009 and others, showed that discrimination for predator $\delta^{15}N$ is prey $\delta^{15}N$ dependent, and used a meta-analytic model to estimate discrimination coefficients.

We initially estimated prey specific discrimination in stable isotopes following Hussey et al 2014, and develop analogous methods for fatty acid signatures. Discrimination is estimated for both types of marker from animals that were fed on diets containing a single prey type (fish or crustaceans). We estimated discrimination coefficients (also termed fractionation or conversion for SI and FA, respectively) using Bayesian models. The analysis can be found in a separate file (Discrimination.Analysis.Rnw or .pdf after compilation) and produces files discr.means.csv/discr.var.csv for SI, and corresponding cc_FA.csv and cc_FA_var.csv for FA.The estimation isn't trivial, so we just pass over it here and refer the interested reader to the separate file. Similarly, we specify fat content using prey sample means and standard deviations to specify log-normal models of prey fat content. The specification is illustrated in a separate file: Squid_prey_fat_cont.Rnw (or .pdf after compilation).

```
## Loading required package:  permute
## Loading required package:  lattice
## This is vegan 2.0-10
## Loading required package:  rjags
## Loading required package:  coda
## Linked to JAGS 3.4.0
## Loaded modules:  basemod,bugs
##
## Attaching package:  'fastinR'
##
## The following objects are masked _by_ '.GlobalEnv':
##
##    dataplot, MCMCplot, pcombine
##
## The following object is masked from 'package:dplyr':
##
##    select_vars
##
## The following object is masked from 'package:utils':
##
##    adist
```

1 Data import

We first read in the data tables, for this we need to be in the Squid example directory in R, or point to the appropriate path. We proceed with the analysis by adding the necessary items for the final analysis to the workspace, using the add_SI and add_FA functions in fastin-R. These functions just add the data, and put them into a specific format.

The FA prey data has 3 profiles for striped mullet - since these are similar to other fish and were fed in very low proportions, we'll exclude them here since we cannot reliably estimate their distributions. We do not have the original fat content data, so we just use the empirical mean and variance to calculate priors for a log-normal model of fat content.

```
# load fatty acid data tables
# prey
prey.ix <- t(read.csv('Prey_FA.csv',header=F,
                      stringsAsFactors=F,row.names=1))[,1]
prey.table.FA <- t(read.csv('Prey_FA.csv',header=T,
                            stringsAsFactors=F,row.names=1))

# remove Striped Mullet from prey index and table
mullets <- which(prey.ix=='Striped Mullet')
```

```r
prey.ix <- prey.ix[-mullets]
prey.table.FA <- prey.table.FA[-mullets,]

# need to (ad-hoc) replace 0 proportions in dataset by min for # that FA
for (i in 1:ncol(prey.table.FA)){
  prey.table.FA[prey.table.FA[,i]==0,i] =
    min(prey.table.FA[prey.table.FA[,i]>0,i])
  }

# cumbersome, but need to add column of prey.ix
prey.table.FA <- data.frame(prey.ix,prey.table.FA)
colnames(prey.table.FA) <- sub('X(*)','\\1',colnames(prey.table.FA))

# predators - subset to Mixed feed only that have SI data (to be quicker)
pred.table.treat <- t(read.csv('Predator_FA.csv',header=T,
                               stringsAsFactors=F,row.names=1))

# switched treatment index - use only samples from specimen fed
# on switched diets for 10 days or more.
SF <- grep('SF',rownames(pred.table.treat))[4:6]
SC <- grep('SC',rownames(pred.table.treat))[4:8]
pred.treat <- c(SF,SC)
pred.treat[1:length(SF)] <- 1
pred.treat[(length(SF)+1):length(pred.treat)] <- 2

# subset the predator table
pred.table.treat <- pred.table.treat[c(SF,SC),]
# same here, replace zeros in FAP
for (i in 1:ncol(pred.table.treat))
  pred.table.treat[pred.table.treat[,i]==0,i] <- min(
    pred.table.treat[pred.table.treat[,i]>0,i])
```

First, we subset the data table for each treatment. Since we only have SI for the same fish for the SC treatment, we analyse SC first with FAP and SI independently to demonstrate the benefit of a combined model. We then use FAP to estimate diets for squid from the SF treatment, and look at a model with group effects to estimate group diet proportions and individual predator diets.

```r
SCs <- grep('SC',rownames(pred.table.treat))
SFs <- grep('SF',rownames(pred.table.treat))

pred.table.treat.SC <- pred.table.treat[SCs,]
pred.table.treat.SF <- pred.table.treat[SFs,]
```

```r
# add data for both treatments
squid.data.treat.SC <- add_FA(FA.predators=pred.table.treat.SC,
                              FA.preys=prey.table.FA,
                              fat.conts='fat.cont.csv',
                              Conv.Coeffs.mean='cc_FA.csv',
                              Conv.Coeffs.var='cc_FA_var.csv',
                              LN.par=T)

squid.data.treat.SF <- add_FA(FA.predators=pred.table.treat.SF,
                              FA.preys=prey.table.FA,
                              fat.conts='fat.cont.csv',
                              Conv.Coeffs.mean='cc_FA.csv',
                              Conv.Coeffs.var='cc_FA_var.csv',
                              LN.par=T)

# a combined object as well
squid.data.treat <- add_FA(FA.predators=pred.table.treat,
                           FA.preys=prey.table.FA,
                           fat.conts='fat.cont.csv',
                           Conv.Coeffs.mean='cc_FA.csv',
                           Conv.Coeffs.var='cc_FA_var.csv',
                           LN.par=T)
```

Plotting the new dataset in MDS-scaled space:

```r
dataplot(squid.data.treat)
```

The NMDS plot (scaled by conversion coefficients) suggests that one group of squid mostly fed on crustaceans, while the other fed mostly on fish - as we would expect.

Instead of using the whole FAP for each predator and prey, we subset:

```r
squid.subset.treat <- select_vars(squid.data.treat)
```

Doing this directly with indices, using the first 4 FAs that combine for $> 69\%$ of separation:

```r
squid.subset.treat.SC <- select_vars(squid.data.treat.SC,
                                     c(24,20,18,10))
squid.subset.treat.SF <- select_vars(squid.data.treat.SF,
                                     c(24,20,18,10))
squid.subset.treat <- select_vars(squid.data.treat,
                                  c(24,20,18,10))
```

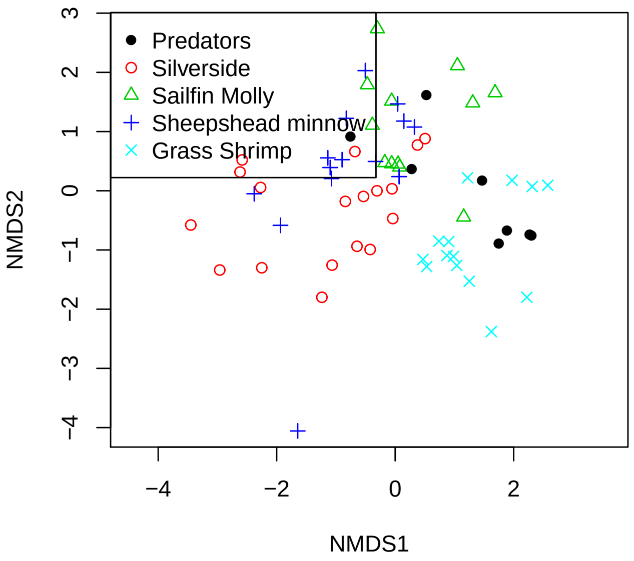

Figure 1: FA projected by NMDS

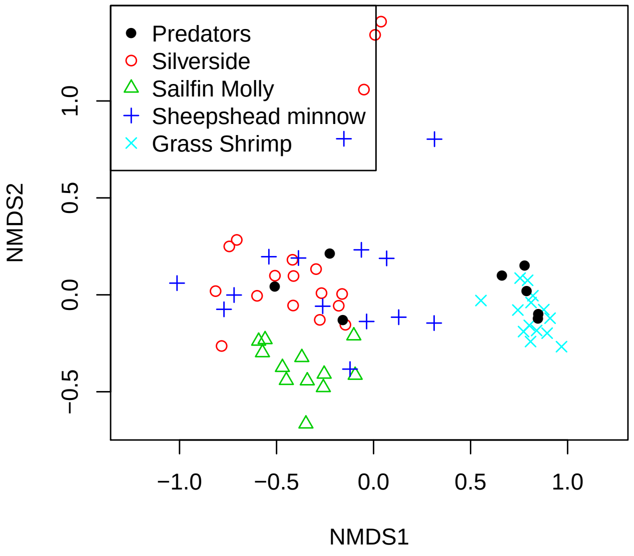

Figure 2: FA subset projected by NMDS

To confirm that we haven't lost too much resolution, we plot the subset in NMDS space:

```
dataplot(squid.subset.treat)
```

This still looks reasonable, which suggests that we can proceed with the analysis. We now can now move to the Bayesian analysis.

2 Bayesian analysis of SC treatment diet composition

2.1 FAP analysis

We start with the default Rnot prior of 0.2, which is reasonably broad and often seems to lead to efficient MCMC sampling (but by no means always!). Prior evenness of diet proportions is set to 0.5, which does constrain extreme proportions somewhat, but often leads to better mixing during the MCMC.

```r
Squid.SC.analysis <- run_MCMC(datas=squid.subset.treat.SC,
                              nIter=600000,
                              nBurnin=10000,
                              nChains=3,
                              nThin=200,
                              Data.Type='Fatty.Acid.Profiles',
                              Analysis.Type='Population.proportions',
                              Rnot=0.2,plott=F,spawn=T)
#diagnostics

diags(Squid.SC.analysis)
```

We evaluate the MCMC visually using coda's plot function, wrapped here to deal with the run_MCMC output class. Note that we don't show the output in the compiled file since it is long (especially for 3 chains). We instead just include the plot below which summarises the results more elegantly.

```r
MCMCplot(Squid.SC.analysis)

summary(Squid.SC.analysis)
```

The summaries show that the posterior distributions are wide for all potential diet items. Nevertheless, as all diagnostics suggest that the chains converged, and the results are in agreement with our expectation, we can now plot the results:

```r
plot(Squid.SC.analysis,save=F,types='post')
```

The posterior suggests a purely shrimp based diet.

2.2 Adding SI

```r
require(dplyr)
prey.table <- read.csv('Prey_SI.csv',header=T,stringsAsFactors=F)
pred.table <- read.csv('Predator_SI.csv',header=T,
                       stringsAsFactors=F)

# use only mixed feed individuals for analysis - using dplyr
pred.table <- pred.table %.%
  filter(Feed=='SC') %.%
```

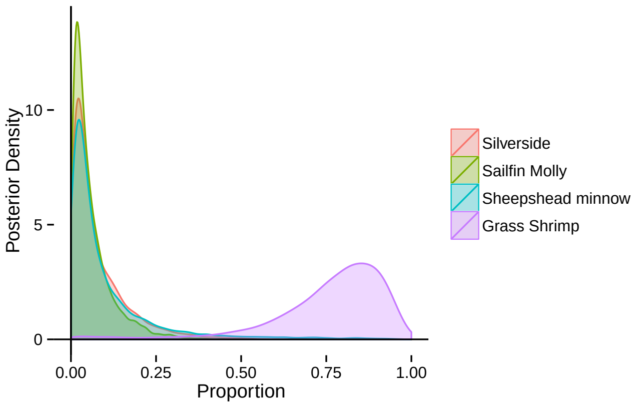

Figure 3: Posterior distributions for diet proportions from FAP markers

```
  select(X_13C,X_15N)

# make sure predators have the same name!
rownames(pred.table) <- rownames(pred.table.treat.SC)
```

We now combine the two data types to a single object, note the datas argument in the function taking the prior data object containing the FAP data:

```
squid.subset.treat.SC.comb <- add_SI(SI.predators=pred.table,
                                     SI.preys=prey.table,
                                     Frac.Coeffs.mean='discr.means.csv',
                                     Frac.Coeffs.var='discr.var.csv',
                                     datas=squid.subset.treat.SC)
```

Looking at a plot of SI alone

```
SID <- squid.subset.treat.comb
asf <- function(x,l) as.numeric(factor(x,levels=l))


mix <- match(SID$prey.ix.SI,
             rownames(SID$datas.SI$mean_cs))

par(mar=rep(5,4))
plot(SID$datas.SI$preys.SI + SID$datas.SI$mean_cs[mix,],
     col = c(rep('#dd4626ff',sum(SID$prey.ix.SI != "Grass shrimp")),
             rep('#2a7fffff',sum(SID$prey.ix.SI == "Grass shrimp"))),
```

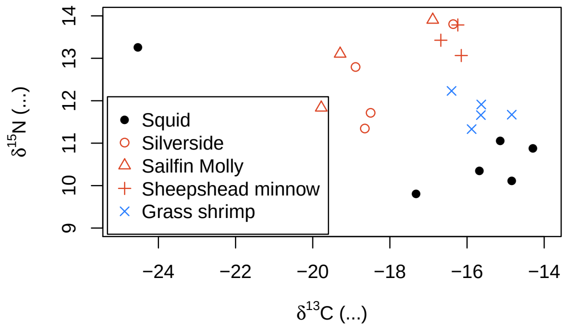

Figure 4: Squid SI

```
     pch = asf(SID$prey.ix.SI,
                  l = unique(SID$prey.ix.SI)),
     ylim = c(9,14),
     xlim= c(-25,-14),
     ylab = expression(paste(delta^{15}, "N (\u2030)")),
     xlab = expression(paste(delta^{13}, "C (\u2030)")))

points(SID$datas.SI$preds.SI,col=1,pch=16)

legend('bottomleft',
       legend = c('Squid',rownames(SID$datas.SI$mean_cs)),
       col = c(1,c(rep('#dd4626ff',3),'#2a7fffff')),
       pch = c(16,asf(rownames(SID$datas.SI$mean_cs),
                      l=unique(SID$prey.ix.SI))),inset = 0.01)
```

Checking the NMDS plot to see what the combined set looks like:

```
dataplot(squid.subset.treat.SC.comb)
```

Curiously, separation seems to be worse, but this may be due to the NMDS not finding a good projection. Dimension reduction can be deceiving if there is no good projection to be found.

```
Squid.SC.SI.analysis <- run_MCMC(datas=squid.subset.treat.SC.comb,
                                 nIter=600000,
                                 nBurnin=10000,
```

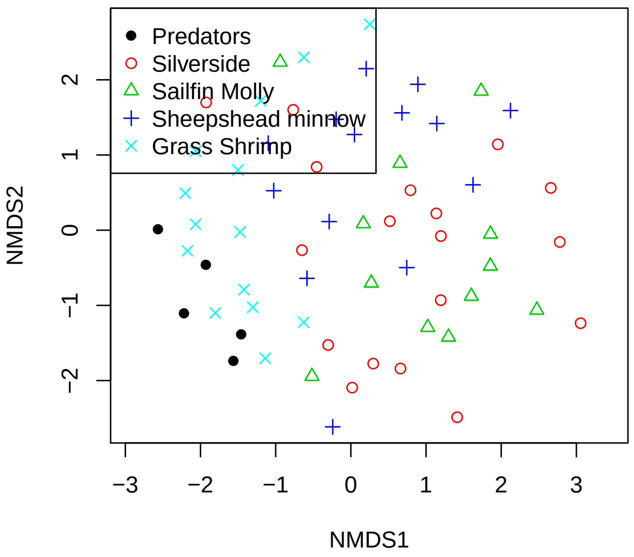

Figure 5: NMDS projection of combined markers

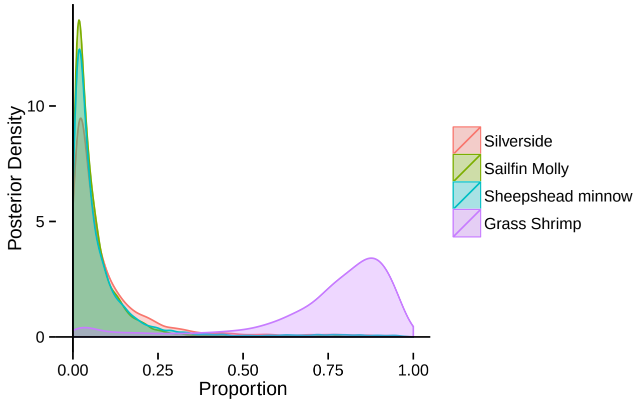

Figure 6: Posterior distributions for diet proportions from SI markers

```
                                    nChains=3,
                                    nThin=200,
                                    Data.Type='Stable.Isotopes',
                                    Analysis.Type='Population.proportions',
                                    Rnot_SI==1,
                                    plott=F, spawn=T)

#diagnostics
diags(Squid.SC.SI.analysis)
```

Again, it looks as though we are close to convergence. Verifying convergence visually and examining summaries of posterior distributions:

```
MCMCplot(Squid.SC.SI.analysis)

#result summary for all 3 chains
summary(Squid.SC.SI.analysis)
```

This looks OK, but would need longer chains and more sub-sampling for a real analysis. The summaries show that the posterior distributions are again very wide, with a 95% density interval of 4% to 96% for shrimp, despite a posterior median that suggests a diet consisting predominantly of shrimp.

```
plot(Squid.SC.SI.analysis,save=F,types='post')
```

In summary, it looks as though both markers can resolve the treatment effect, but leave a long tail in the posterior that equates to uncertainty about SC diet proportions. A combined analysis of FAP and SI provides a clearer picture:

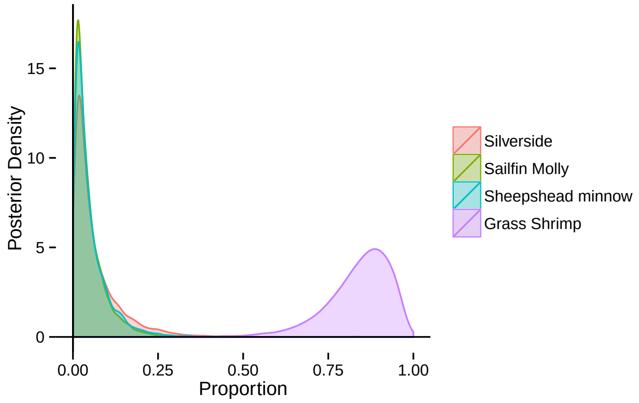

Figure 7: Posterior distributions for diet proportions from combined markers

2.3 Combined SI and FAP analysis

```
Squid.SC.comb.analysis <- run_MCMC(datas=squid.subset.treat.SC.comb,
                                   nIter=600000,
                                   nBurnin=10000,
                                   nChains=3,
                                   nThin=200,
                                   Data.Type='Combined.Analysis',
                                   Analysis.Type='Population.proportions',
                                   Rnot=0.2, Rnot_SI=0.2,
                                   plott=F, spawn=T)

#diagnostics
diags(Squid.SC.comb.analysis)
```

Making sure that the chains converged, and looking at posterior summaries.

```
MCMCplot(Squid.SC.comb.analysis)

summary(Squid.SC.comb.analysis)
```

Estimates of diet proportion from all three chains converge to consistent values, with a posterior median of 87% shrimp, and a 95% interval of 67% - 96.5%. Plotting the final combined proportions:

```
plot(Squid.SC.comb.analysis,save=F,types='post')
```

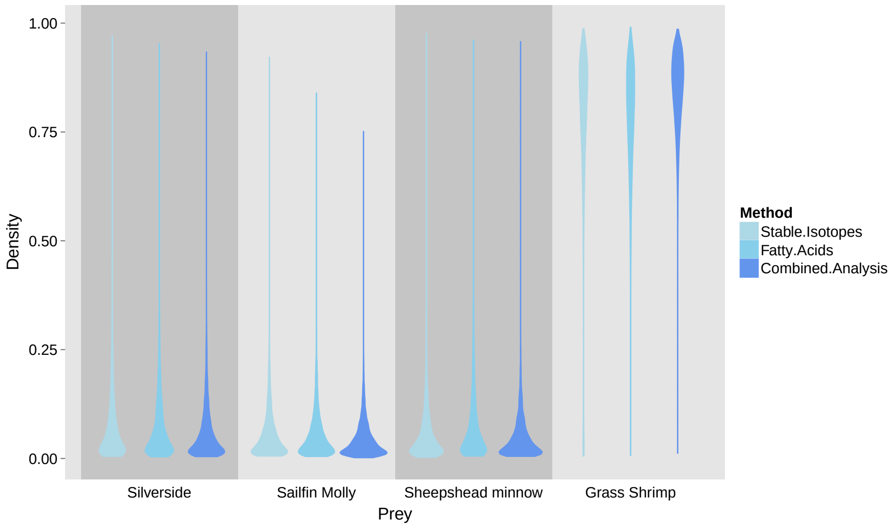

Figure 8: Comparison of posterior distributions for diet proportions from FA, SI and combined markers

We can directly compare population level proportions using the *multiplot* function.

```
# combining individual elements to a named list
Pop.list <- list(
  Stable.Isotopes = Squid.SC.SI.analysis,
  Fatty.Acids = Squid.SC.analysis,
  Combined.Analysis = Squid.SC.comb.analysis)

#plotting
multiplot(Pop.list,save=F,types='violin')
```

This combined plot illustrates the advantage of using the two markers together, reducing uncertainty and obtaining better point estimates.

Now that we have some confidence that we can resolve overall proportions, what about individual estimates?

2.4 How sensitive are results to FA subsets?

We only used four fatty acids in the analysis so far. What if we chose more, leading to potentially better separation among species?

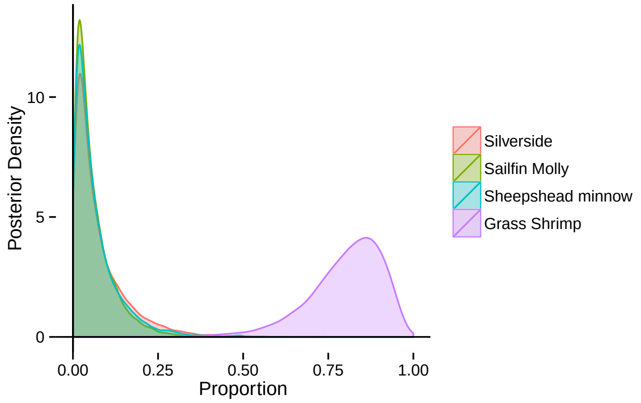

Figure 9: Posterior distributions for diet proportions from FA markers with larger FA subset

```
squid.subset.treat.SC.alt <- select_vars(squid.data.treat.SC,c(24,20,18,10,23,5,12,11))
```

```
Squid.SC.analysis.alt <- run_MCMC(datas=squid.subset.treat.SC.alt,
                                  nIter=600000,
                                  nBurnin=10000,
                                  nChains=3,
                                  nThin=200,
                                  Data.Type='Fatty.Acid.Profiles',
                                  Analysis.Type='Population.proportions',
                                  Rnot=0.2,plott=F,spawn=T)
#diagnostics

diags(Squid.SC.analysis.alt)
```

Looking at MCMC chains and output summaries:

```
MCMCplot(Squid.SC.analysis.alt)

summary(Squid.SC.analysis.alt)
```

The summary suggests that we can get tighter confidence intervals by including more informative FAs. Plotting this gives:

```
plot(Squid.SC.analysis.alt,save=F,types='post')
```

Does the combined analysis still provide a benefit over FAP alone? We need to add SI data to the new FAP data object:

```
squid.subset.treat.SC.comb.alt <- add_SI(SI.predators=pred.table,
                                     SI.preys=prey.table,
                                     Frac.Coeffs.mean='discr.means.csv',
                                     Frac.Coeffs.var='discr.var.csv',
                                     datas=squid.subset.treat.SC.alt)
```

Now re-run the combined analysis:

```
Squid.SC.comb.analysis.alt <- run_MCMC(datas=squid.subset.treat.SC.comb.alt,
                                   nIter=600000,
                                   nBurnin=10000,
                                   nChains=3,
                                   nThin=200,
                                   Data.Type='Combined.Analysis',
                                   Analysis.Type='Population.proportions',
                                   Rnot=0.2, Rnot_SI=0.2,
                                   plott=F, spawn=T)

#diagnostics
diags(Squid.SC.comb.analysis.alt)
```

Making sure that the chains converged, and looking at posterior summaries.

```
MCMCplot(Squid.SC.comb.analysis.alt)

summary(Squid.SC.comb.analysis.alt)
```

We can again compare population level proportions between approaches using the *multiplot* function.

```
# combining individual elements to a named list
Pop.list.alt <- list(
  Fatty.Acids = Squid.SC.analysis.alt,
  Combined.Analysis = Squid.SC.comb.analysis.alt)

#plotting
multiplot(Pop.list.alt,save=F,types = 'violin')
```

The answer of narrower intervals and lower uncertainty when combining markers for this analysis seems to be valid despite the increased information content in FAPs alone.

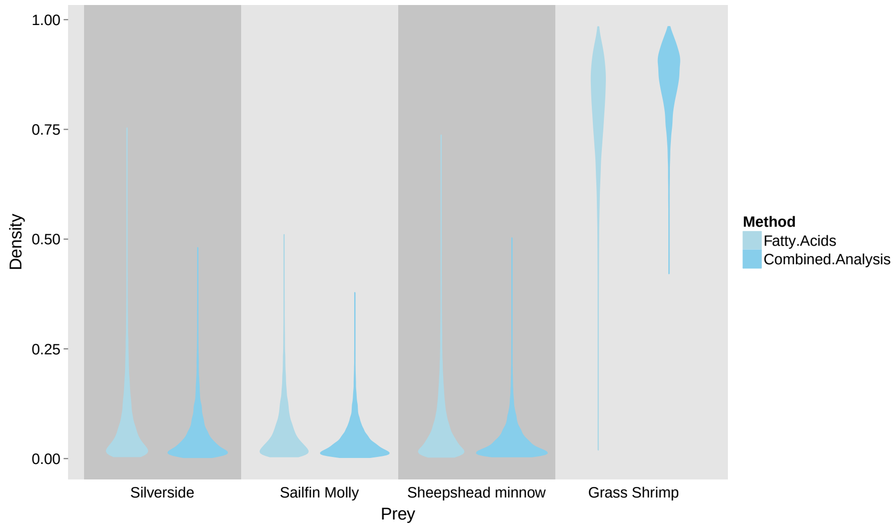

Figure 10: Comparison of posterior distributions for diet proportions from FA and combined markers

2.5 Analysing individual diet proportions

```
Squid.SC.analysis.ind <- run_MCMC(datas=squid.subset.treat.SC.comb,
                                  nIter=600000,
                                  nBurnin=10000,
                                  nChains=3,
                                  nThin=200,
                                  Data.Type='Combined.Analysis',
                                  Analysis.Type='Individual.proportions',
                                  Rnot=0.1, even=0.1, Rnot_SI=0.2,
                                  plott=F, spawn=T)


#diagnostics
diags(Squid.SC.analysis.ind)
```

It looks as though the chains could have run longer than we did here, but MCMC plots show reasonable convergence.

```
MCMCplot(Squid.SC.analysis.ind)

summary(Squid.SC.analysis.ind)
```

Estimates of population level diet proportion converge to a posterior median of 90% shrimp, and a 95% credible interval of 42% - 99%. Individual estimates are very similar (not surprisingly...). Plotting the final combined proportions:

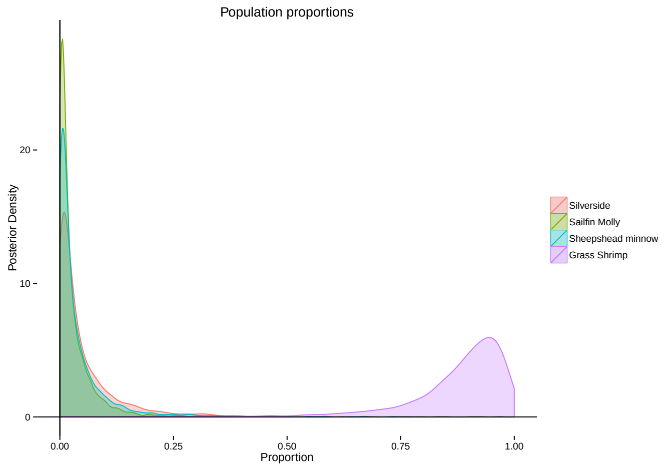

Figure 11: Population and individual posterior distributions of diet proportions from combined markers

```
plot(Squid.SC.analysis.ind,save=F,types='post')
```

Again, it looks like all of the squid ate only shrimp, with individual posterior distributions for all fish species peaking at zero for all predators.

3 FAP analysis of SF

For a population level analysis, we do the same as for SC squid before. Starting with just FAP.

```
Squid.SF.analysis <- run_MCMC(datas=squid.subset.treat.SF,
                              nIter=600000,
                              nBurnin=10000,
                              nChains=3,
                              nThin=200,
                              Data.Type='Fatty.Acid.Profiles',
                              Analysis.Type='Population.proportions',
                              Rnot=0.02,
                              plott=F, spawn=T)

#diagnostics
diags(Squid.SF.analysis)
```

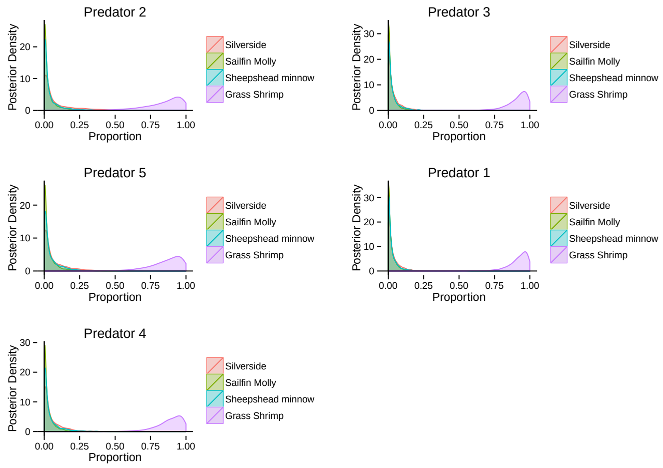

Figure 12: Population and individual posterior distributions of diet proportions from combined markers

```
MCMCplot(Squid.SF.analysis)

summary(Squid.SF.analysis)
```

```
plot(Squid.SF.analysis,save=F)
```

Again, the results are in line with expectations, although the posterior distributions for the individual fish species all show long tails, and Silverside in particular shows a very broad and bimodal posterior distribution, leaving their relative contribution somewhat uncertain. Furthermore, there is still high uncertainty regarding the proportion of crustaceans in the diet. This is not surprising given the overlap in NMDS space visible on the plots above, meaning that we can't really tell which fish species contributed.

3.0.1 Adding SI

```
detach("package:dplyr", unload=TRUE)
require(dplyr)
prey.table <- read.csv('Prey_SI.csv',header=T,
                       stringsAsFactors=F)
pred.table <- read.csv('Predator_SI.csv',header=T,
                       stringsAsFactors=F)

# use only mixed feed individuals for analysis - using dplyr
```

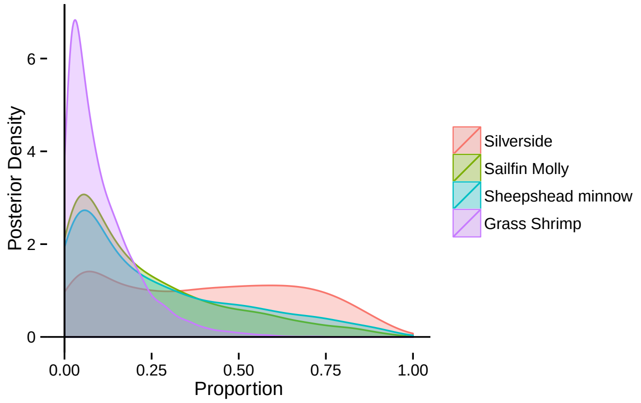

Figure 13: Posterior distributions of diet proportions for SF treatment Squid from FA markers

```
pred.table <- pred.table %.%
  filter(Feed=='SF') %.%
  select(X_13C,X_15N)
```

We now combine the two data types to a single object, note the datas argument in the function taking the prior data object containing the FAP data:

```
squid.subset.treat.SF.comb <- add_SI(SI.predators=pred.table,
                                     SI.preys=prey.table,
                                     Frac.Coeffs.mean='discr.means.csv',
                                     Frac.Coeffs.var='discr.var.csv',
                                     datas=squid.subset.treat.SF)
```

Since we don't have overlapping data, it will be interesting to see if the two markers can improve population level estimates.

```
Squid.SF.comb.analysis <- run_MCMC(datas=squid.subset.treat.SF.comb,
                                   nIter=600000,
                                   nBurnin=10000,
                                   nChains=3,
                                   nThin=200,
                                   Data.Type='Combined.Analysis',
                                   Analysis.Type='Population.proportions',
                                   Rnot=0.2, Rnot_SI=0.2,
                                   plott=F, spawn=T)
```

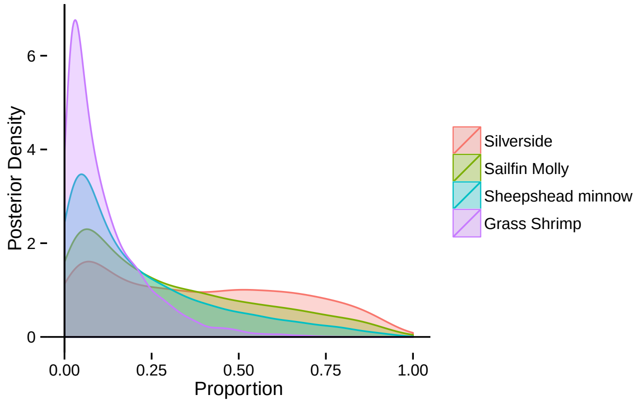

Figure 14: Posterior distributions of diet proportions for SF treatment squid from combined markers

```
#diagnostics
diags(Squid.SF.comb.analysis)
```

Making sure that the chains converged, and looking at posterior summaries.

```
MCMCplot(Squid.SF.comb.analysis)

summary(Squid.SF.comb.analysis)
```

Estimates of diet proportion from all three chains converge to consistent values, with higher proportions for fish than for shrimp, but still considerable uncertainty for all potential diets:

```
plot(Squid.SF.comb.analysis,save=F,types='post')
```

```
Squid.fish.com <- pcombine(Squid.SF.comb.analysis,1:3)
plot(Squid.fish.com,save=F)
```

We can again directly compare population level proportions using the *multiplot* function.

```
# combining individual elements to a named list
Pop.list.SF <- list(
  Fatty.Acids = Squid.SF.analysis,
  Combined.Analysis = Squid.SF.comb.analysis)
```

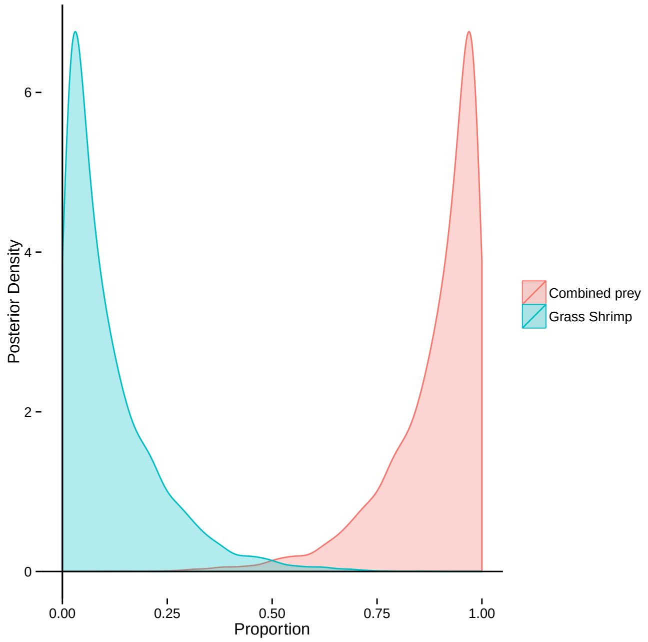

Figure 15: Posterior distributions of diet proportions for SF treatment Squid from combined markers, with fish species combined

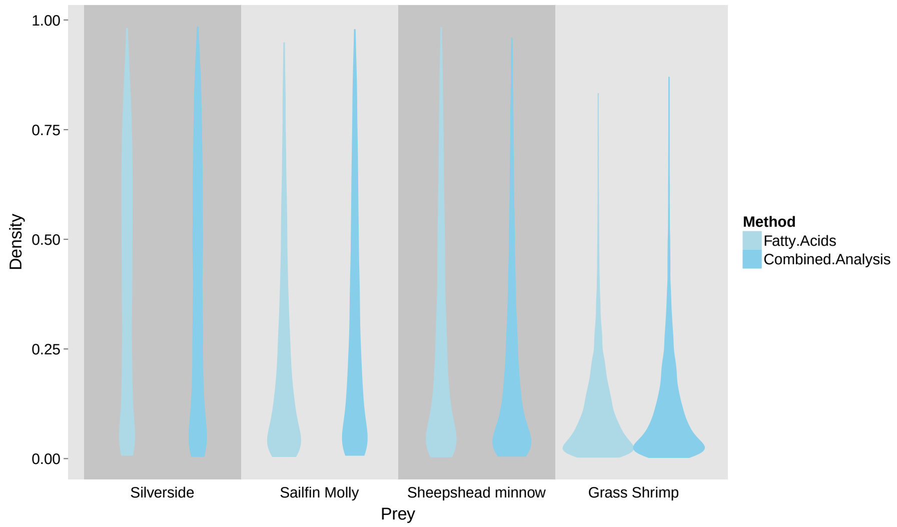

Figure 16: Posterior distributions of diet proportions for SF treatment squid from combined markers, grouping fish species post MCMC

```
#plotting
multiplot(Pop.list.SF,save=F,types='violin')
```

Estimating individual proportions for this treatment is hard, because of the overlap of fish species in FA space. We'll try anyway, combining with the SC treatment and adding a group effect for a complete analysis that estimates treatment effects and group specific population level diets:

4 Combined analysis of treatment effects

```
detach("package:dplyr", unload=TRUE)
require(dplyr)
prey.table <- read.csv('Prey_SI.csv',header=T,
                       stringsAsFactors=F)
pred.table <- read.csv('Predator_SI.csv',header=T,
                       stringsAsFactors=F)

# use only mixed feed individuals for analysis - using dplyr
pred.table <- pred.table %.%
  filter(Feed=='SC'|Feed=='SF') %.%
  select(X_13C,X_15N)
```

```r
# make sure predators have the same name!
rownames(pred.table)[-1] <- rownames(pred.table.treat.SC)
rownames(pred.table)[1] <- 'SF.1'

#making a combined object with both data types;
squid.subset.treat.comb <- add_SI(SI.predators=pred.table,
                                  SI.preys=prey.table,
                                  Frac.Coeffs.mean='discr.means.csv',
                                  Frac.Coeffs.var='discr.var.csv',
                                  datas=squid.subset.treat)

treat <- as.numeric(grepl('SC',rownames(squid.subset.treat.comb$datas.SI$preds.SI)))
# add group dummy variables
treatments <- add_Covs(Groups=as.data.frame(treat))
```

```r
Squid.treat.analysis.comb <- run_MCMC(datas=squid.subset.treat.comb,
                                      Covs=treatments,nIter=600000,
                                      nBurnin=50000,
                                      nChains=3,
                                      nThin=200,
                                      Data.Type='Combined.Analysis',
                                      Analysis.Type='Analysis.with.Covariates',
                                      Rnot=0.2, even=0.1,Rnot_SI=0.2,
                                      plott=F, spawn=T)

#diagnostics
diags(Squid.treat.analysis.comb)
```

```r
MCMCplot(Squid.treat.analysis.comb)

summary(Squid.treat.analysis.comb)
```

The group level contrasts in the summary suggest that we cannot reliably tell if for SC treatment squid the ratio of fish to grass shrimp consumed is significantly lower than for SF treatment squid. This is due to uncertainty for the contribution of individual fish species at the SF treatment level.

```r
plot(Squid.treat.analysis.comb,save=F,types = 'post')
```

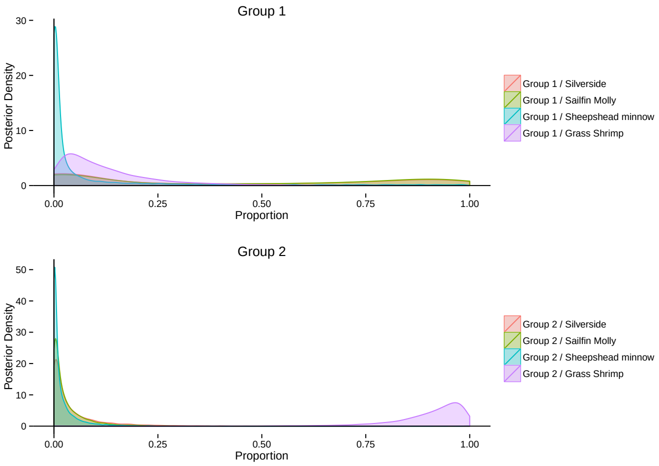

Figure 17: Posterior distributions at the popualtion level for feeding groups and at the individual level for individual squid from all treatments using combined markers

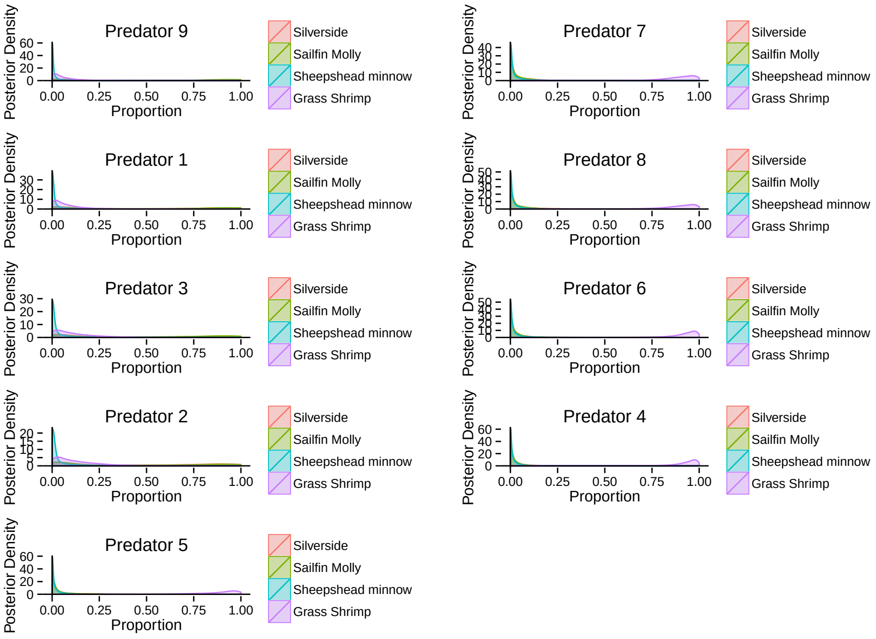

Figure 18: Posterior distributions at the popualtion level for feeding groups and at the individual level for individual squid from all treatments using combined markers
